# Pseudo-Temporal Analysis of Single-Cell RNA Sequencing Reveals *Trans*-Differentiation Potential of Greater Epithelial Ridge Cells Into Hair Cells During Postnatal Development of Cochlea in Rats

**DOI:** 10.3389/fnmol.2022.832813

**Published:** 2022-03-16

**Authors:** Jianyong Chen, Dekun Gao, Junmin Chen, Shule Hou, Baihui He, Yue Li, Shuna Li, Fan Zhang, Xiayu Sun, Yulian Jin, Lianhua Sun, Jun Yang

**Affiliations:** ^1^Department of Otorhinolaryngology-Head and Neck Surgery, Xinhua Hospital, Shanghai Jiao Tong University School of Medicine, Shanghai, China; ^2^Institute of Ear Science, School of Medicine, Shanghai Jiao Tong University, Shanghai, China; ^3^Shanghai Key Laboratory of Otolaryngology and Translational Medicine, Shanghai, China

**Keywords:** sc-RNA sequencing, pseudo-temporal analysis, cochlear basal membrane, greater epithelial ridge cells, hair cells

## Abstract

The hair cells of the cochlea play a decisive role in the process of hearing damage and recovery, yet knowledge of their regeneration process is still limited. Greater epithelial ridge (GER) cells, a type of cell present during cochlear development that has the characteristics of a precursor sensory cell, disappear at the time of maturation of hearing development. Its development and evolution remain mysterious for many years. Here, we performed single-cell RNA sequencing to profile the gene expression landscapes of rats’ cochlear basal membrane from P1, P7, and P14 and identified eight major subtypes of GER cells. Furthermore, single-cell trajectory analysis for GER cells and hair cells indicated that among the different subtypes of GER, four subtypes had transient cell proliferation after birth and could transdifferentiate into inner and outer hair cells, and two of them mainly transdifferentiated into inner hair cells. The other two subtypes eventually transdifferentiate into outer hair cells. Our study lays the groundwork for elucidating the mechanisms of the key regulatory genes and signaling pathways in the *trans*-differentiation of GER cell subtypes into hair cells and provides potential clues to understand hair cell regeneration.

## Introduction

In the mammalian auditory system, irreversible damage to cochlear hair cells can be caused by noise stimulation, aging, ototoxic drugs, infections, hereditary susceptibility, and autoimmune diseases, resulting in permanent sensorineural deafness (Wagner and Shin, 2019). Studies have shown that in lower vertebrates, hair cells can regenerate after damage (Cruz et al., 1987), but hair cell regeneration has not been achieved in the mature mammalian cochlea (Brigande and Heller, 2009). The damage and loss of cochlear hair cells are still an important cause of mammalian auditory injury.

The Corti’s organ is composed of a certain number of sensory hair cells and supporting cells arranged in a highly ordered manner into a precise chimeric structure (Lim, 1986). The hair cells are located on the basal membrane of the Corti’s organ and are closely connected to the non-sensory epithelial cells, which are the main components of the auditory pathway. Hair cells are divided into inner and outer hair cells. Supporting cells are composed of several different types, including inner and outer pillar cells, Deiters cells, Hensen cells, Claudius cells, inner phalangeal cells, and inner border cells. The heads of the inner and outer pillar cells are articulated, and the bases are separated to form a triangular spiral tunnel. Inside the tunnel, there is a single row of inner hair cells on the inner phalangeal cells inside the inner pillar cells. Between the inner phalangeal cells and the inner hair cell is the inner border cell, and its inner side is the inner sulcus. The bottom cell of the inner sulcus has a single layer of cubic epithelium, which is the inner sulcus cell, which comes from the greater epithelial ridge (GER) (Lim and Anniko, 1985).

Histological studies revealed that progenitor cells of the rodent cochlear epithelium develop from the auditory vesicles at embryonic day (E) 11.5, and subsequently proliferate and expand into medial GER and lateral lesser epithelial ridge (LER). From about E15 to birth, GER and LER gradually differentiate into hair cells and supporting cells of the primitive Corti’s organ (Driver and Kelley, 2020). Zheng and Gao (2000) transfected a math1-expressing plasmid into the cochlea of newborn rats and found that myosin VIIa (a specific marker of hair cells) and peanut agglutinin-labeled hair cells were produced in the GER region. The origin of these ectopic hair cells is thought to be columnar epithelial cells located in GER.

Greater epithelial ridge normally exists in the late embryonic and early postnatal stages and is located around the inner hair cells, which is one of the signs of immature cochlear morphology (Hinojosa, 1977). After birth, the cell structure rearranges, the number of cells decreases, and the cells degenerate and disappear to become internal sulcus cells around 10–12 days, and eventually form the inner sulcus, at which point the auditory development of cochlea is mature (Kelley, 2007; Dayaratne et al., 2014). The emergence of ectopic hair cells is a surprising discovery since the precursor cells of hair cells were previously thought to originate from the sensory epithelium, but the columnar epithelial cells in GER are located outside of the sensory epithelium, so the researchers considered that GER cells were likely to be the precursor cell pool for hair cell regeneration in the cochlea (Zine et al., 2001). At the same time, a study using adenoviral vectors to transfect genes into the cochlea of mature guinea pigs showed that immature hair cells were generated in the area where GER was located and connected to auditory neurons, further demonstrating that GER was probably a precursor cell for cochlear hair cells (Kawamoto et al., 2003). In addition, organoid developmental regeneration studies showed that different types of non-sensory epithelial cells had the potential for organoid regeneration and development and that organoids derived from GER cells contained cells positive for hair cell markers, suggesting that GER cells had the characteristics of precursor sensory cells after mitosis (Kubota et al., 2021).

Single-cell transcriptome sequencing technology has shown that there are different cell subtypes within the GER region, and different cell subtypes have heterogeneity of gene expression and diversity of biological functions (Kolla et al., 2020; Chen et al., 2021). At present, which GER subtype may transform into inner hair cells and which GER subtype may transform into outer hair cells has not been reported in the literature. In this study, we investigated the differentiation trajectories of different subtypes of GER by single-cell trajectory analysis. The results showed that among the different subtypes of GER, four subtypes had transient cell proliferation after birth and could transdifferentiate into inner and outer hair cells, and two of them mainly transdifferentiated into inner hair cells. The other two subtypes eventually transdifferentiate into outer hair cells. In this study, we revealed the key regulatory genes and signaling pathways in the *trans*-differentiation of GER cell subtypes into hair cells, providing new ideas for the study of the influencing factors and mechanisms of hair cell differentiation and regeneration.

## Materials and Methods

### Tissue Preparation

Female and/or male Sprague–Dawley (SD) rats were selected for this study, which was purchased from Shanghai SIPPR-BK Laboratory Animal Co. In this study, The P1 means the first postnatal day, the P7 day refers to the seventh day after birth, and P14 is the fourteenth postnatal day. Forty SD rats were randomly selected for each period. The animal experiments were performed following the ethical requirements approved by the Animal Care and Use Committee of Shanghai Jiao Tong University School of Medicine. To obtain fresh cochlear basal membrane tissue, the approved guillotine method was used. Cochlear basal membrane tissue was isolated from the temporal bone using a microdissection technique in cold RNase-free Hank’s Balanced Salt Solution (HBSS), and the ear capsule was carefully transferred to a tray containing 0.01 M cold phosphate-buffered sodium saline (PBS, pH 7.35, GIBCO, Invitrogen Inc., Carlsbad, CA, United States). The spiral ganglia, spiral ligaments, and vascular striae were carefully separated from the cochlear basal membrane, and the isolated cochlear basal membrane was washed twice with PBS without potassium and magnesium.

### Preparation of Single-Cell Suspensions

Cochlear basal membrane tissues were removed *ex vivo* and placed in pre-chilled sterile PBS (calcium-free and magnesium-free) solution, then washed and cut into 0.5 mm^2^ pieces. The tissue was dissociated into single cells using a dissociation solution (0.35% collagenase IV5, 2 mg/ml papain, 120 units/ml DNase I) in a 37°C water bath, and digestion was terminated with PBS containing 10% fetal bovine serum. The acquired cell suspension was filtered through a 70–30 um filter and centrifuged for 5 min (4°C, 300 g). The cell precipitate was resuspended in 100 ul of PBS (0.04% BSA) and 1 ml of erythrocyte lysis buffer (MACS 130-094-183) was added and the remaining erythrocytes were lysed by incubation at room temperature for 10 min. After centrifugation again, the precipitate is suspended in 100 μl of Dead Cell Removal Microbeads (MACS 130-090-101) and the dead cells are removed using the Miltenyi^®^ Dead Cell Removal Kit (MACS 130-090-101). The cells were suspended in PBS (0.04% BSA) after repeated centrifugation and resuspension twice.

Cell viability was checked by trypan blue assay to ensure it was above 85%, single-cell suspensions were counted using a hematocrit plate and the concentration was adjusted to 700–1200 cells/μl.

### Chromium 10× Genomics Library and Sequencing

Single-cell suspensions were loaded into 10× Chromium according to kit instructions to capture 5000 single cells, followed by cDNA amplification and library construction according to standard procedures. Sequencing was performed using an Illumina NovaSeq 6000 sequencing system (paired-end multiplex run, 150 bp) by LC-Bio Technology Co. Ltd. (Hangzhou, China) with a minimum depth of 20,000 reads per cell.

### Bioinformatics Analysis of scRNA-Seq Data

Illumina bcl2fastq software was used to demultiplex and convert sequencing results to FASTQ data format. Sample demultiplexing, barcode processing and single-cell 3′ gene counting by using the Cell Ranger pipeline^1^ and sc-RNA-seq data were aligned to Rattus norvegicus reference genome (Source: Rattus norvegicus UCSC; version: rn6), Single-cells were processed using 10× Genomics Chromium Single Cell 3′ Solution was used to process the captured single cell (Baslan et al., 2012; Shapiro et al., 2013; Huang et al., 2018). The Cell Ranger output was loaded into Seurat (version 3.1.1) to be used for dimensional reduction, clustering, and analysis of scRNA-sequencing data. A total of 34,927 cells, in the end, passed quality control: all genes expressed in less than 1 cell were removed, the number of genes expressed per cell >500 as low and <5000 as high cut-off, UMI counts <500, and the percentage of mitochondria-DNA-derived genes expressed was <25%. The percent of mitochondrial-DNA-derived gene-expression <25%.

### Identification of the Major Cell Types and Their Subtypes

We used Seurat software to reduce the dimensionality of all 34,927 cells to visualize them and projected them into two dimensions using the t-SNE method (Satija et al., 2015), with the following steps briefly described: (1) Calculation of gene expression values using the LogNormalize method in Seurat software; (2) Dimensionality reduction of data was performed by using PCA (Principal component analysis) based on the first 2000 highest variable genes. Within all the PCs, the top 10 PCs were used to do clustering and t-SNE analysis; (3) To find clusters, the Seurat Find Clusters function is used to divide all cells into different cell populations with a resolution of 0.8, and clustering results were visualized by using t-distributed Stochastic Neighbor Embedding (tSNE); (4) Marker genes for each cluster were identified with the Wilcoxon rank-sum test with default parameters *via* the FindAllMarkers function in Seurat.

### Trajectory and Pseudotime Analysis

CellTrails and Monocle 2^2^ (Trapnell et al., 2014) software were used to perform trajectory and pseudotime analysis. We first identified genes that changed over time using a previous study (Yee, 2004) and then performed a likelihood ratio test analysis of differential genes using the differential gene test function to identify significant genes with FDR less than 0.05. Finally, the genes were clustered into groups using the pam function in the cluster R package, and cell sorting and trajectory construction were performed on these genes in an unsupervised manner.

### Trajectory Gene Dynamics and Differential Gene Expression

To identify genes whose expression is significantly altered during cochlear development, we took advantage of the fit Dynamic function in CellTrails. We extracted the developmental trajectory of each cluster from the branching trajectory map and calculated the association of gene expression using the R mgcv package.^3^ The derived gene sets were then further characterized by the AnimalTFDB 3.0 database containing 1,636 transcription factors (Hu et al., 2019). Finally, dynamic differences in expression between genes were determined using the contrastTrailExpr function. The root means square deviation (RMSD) was used to estimate the difference between trail expressions.

### Cell State Identification

The CellTrails package^4^ uses spectral embedding and hierarchical clustering methods to identify the state of cell clusters (Ellwanger et al., 2018) and to infer the topology of developmental trajectories. The approach first identifies the most obvious variable genes using the method of M3Drop^5^ and then identifies scRNA-Seq datasets using the Michaelis–Menten model (Andrews and Hemberg, 2019). M3Drop identifies differentially expressed genes at 5% FDR and clusters were visualized in a force-based layout, based on log ratios of high-weight paths between clusters. According to the principle of FDR less than 1%, the Wilcoxon rank-sum test was used to identify differentially expressed genes or marker genes among cell clusters.

### Pathway Enrichment Analysis

Gene Ontology (GO) enrichment analysis was performed using the GO package (Brionne et al., 2019) and Kyoto Encyclopedia of Genes and Genomes (KEGG) pathway enrichment analysis was performed using Ingenuity Pathway Analysis (IPA) (Krämer et al., 2014) to examine enrichment in clusters processes. Both analyses allow us to determine which GO terms and/or metabolic pathways are significantly enriched during development. Adjusted *p*-values < 0.05 were considered statistically significantly different.

### Cell-Cell Communication Analysis

To investigate the potential interactions between different cell subpopulations in GER cells, inner and outer hair cells, CellPhoneDB was used for communication analysis (Dou et al., 2021). First, pairwise comparisons were performed between the cell clusters included in the analysis. The cluster labels of all cells were first randomized 1000 times to determine the average receptor and ligand expression levels for each interacting cell cluster. A null distribution for each receptor-ligand pair was generated. The probability of cell-type specificity of the corresponding receptor-ligand complex was obtained by calculating the proportion of the mean higher than the actual mean to derive a *P*-value. Finally, biologically relevant interactions are derived.

### Fluorescence *in situ* Hybridization to Verify Gene Expression Changes

The localization of gene expression and dynamic changes at postnatal day 1 (P1), day 7 (P7), and day 14 (P1) were verified by Paraffin-DIG (digoxigenin)-TSA (Tyramine Signal Amplification)-ISH protocol. Cochlear basal membranes of P1, P7, and P14 SD rats of both sexes were collected and fixed in 4% paraformaldehyde overnight. The Cochleae tissues were then dehydrated with graded alcohol, paraffinized, embedded, and sectioned at a 10-μm thickness on a cryostat. The hybridization protocol was performed according to the manufacturer’s recommendations. After RNA ISH, sections were washed with 2 × SSC for 10 min at 37°C, twice with 1 × SSC for 5 min, and then with 0.5 × SSC for 10 min at room temperature. If more non-specific hybrids appear, formamide can be added for washing. Blocking solution (rabbit serum) was added to the section and incubated at room temperature for 30 min, and then remove the blocking solution and add anti-digoxigenin-labeled peroxidase. The sections were incubated at 37 °C for 40 min and then washed with PBS four times for 5 min each. Nuclei were counterstained with DAPI for 15 s at room temperature. After that, a Nikon Eclipse CI upright fluorescence microscope was used to obtain all fluorescent images.

### Statistical Analysis

All statistical analyses of the cochlear cells data described in this paper were performed using Prism version 7.0 (GraphPad Software) and calculated according to the relative abundances. Experimental data are presented as the mean ± SEM. Comparisons were made by one-way analyses of variance or students’ unpaired two-tailed *t*-tests and unpaired Wilcoxon rank-sum test among three different stages. *P* values were calculated using a two-tailed Student’s *t*-test, and *P* values < 0.05 were considered statistically significant differences.

## Results

### scRNA-Seq Identifies Eight Greater Epithelial Ridge Cell Clusters According to the Cells Number Dynamic Change and Gene Expression for Significant Marker Genes During Postnatal Auditory Development in Rats

**FIGURE 1:**
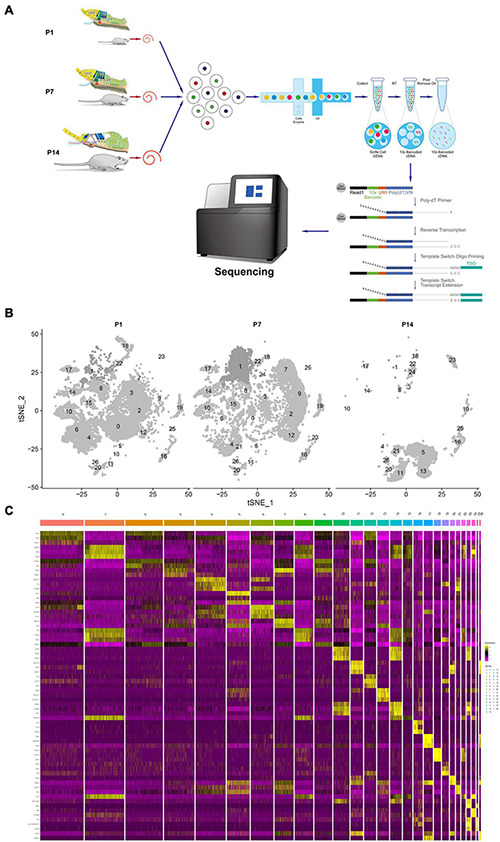
Global expression profiling of cochlear duct cells by scRNA-Seq and cell clusters identification from P1, P7, and P14. **(A)** Cross-section diagrams of the cochlear canals, and the scheme of cochlear duct preparation, single-cell isolation, and Chromium 10× Genomics library and scRNA-seq at P1, P7, and P14. **(B)** t-SNE plots of cochlear cell clusters at P1 (left), P7 (middle), and P14 (right) based on the origins (number) are shown. **(C)** Heat map for cochlear duct cell clusters. The top three differentials expressed (DE) genes for the 27 identified clusters are shown. Cellular identity for each cluster is indicated by a color bar at the top of the heat map. The color ranges from blue to bright yellow indicates low to high gene expression levels, respectively.

Single-cell RNA sequencing technology was used to perform transcriptome analysis of rat cochlear basal membrane at three critical periods (P1, P7, and P14), and cells with the same gene expression pattern were clustered together according to the cellular gene expression pattern (Figure 1A). After sequencing analysis, we constructed cell profiles and identified 27 cell clusters in the three periods (Figure 1B). We can see that the number of cells in some clusters decreased significantly or even disappeared at day P14, some clusters were present in increased numbers at day P7 and then decreased significantly and disappeared further by day P14, and some clusters showed no significant changes. The gene expression heat map shows the top three expressed genes for each different cell cluster (Figure 1C and Supplementary Table 1).

**FIGURE 2:**
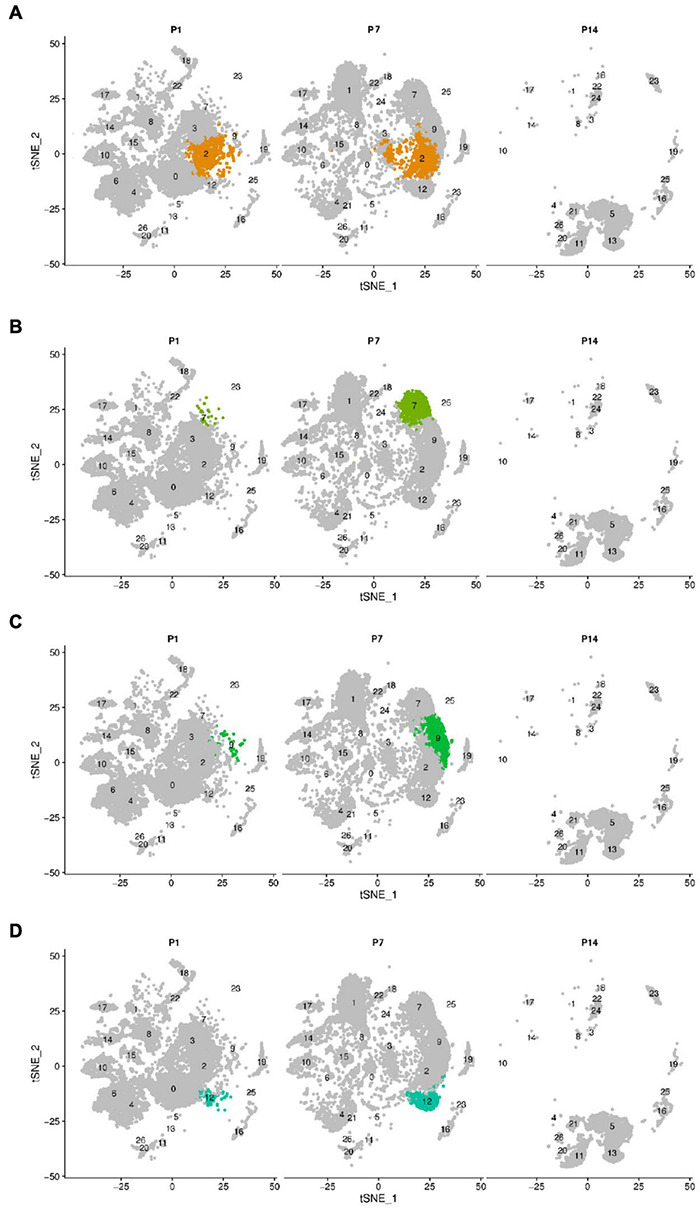
The change of cell number of the above different cell clusters in three different periods of P1, P7, and P14. Cells were clustered using a graph-based shared nearest-neighbor clustering approach plotted by a tSNE plot. **(A)** tSNE plot of Cluster 2. **(B)** tSNE plot of Cluster 7. **(C)** tSNE plot of Cluster 9. **(D)** tSNE plot of Cluster 12. Clusters 2, 7, 9, and 12 showed an increase in the number of cells from P1 to P7, while degenerated and disappeared at P14 days.

Greater epithelial ridge cells, also referred to as Kölliker’s organ (KO) (Dou et al., 2021) are temporary structures during the development of cochlear hearing, during which there is programmed apoptosis and autophagy, and they eventually degenerate and disappear after the cochlear hearing has matured (Hou et al., 2019, 2020). From the dynamic change analysis of cell numbers, four-cell clusters, 0, 3, 4, and 6, showed a gradual decrease in cell numbers from P1 to P14 and disappeared on P14, and we tentatively considered four cell clusters 0, 3, 4, and 6 as different subtypes of GER cells (Chen et al., 2021), which is consistent with Kolla’s study (Kolla et al., 2020). In addition, we also found that clusters 2, 7, 9, and 12 showed an increase in the number of cells from P1 to P7, while degenerated and disappeared at P14 days (Figure 2 and Supplementary Table 1).

**FIGURE 3:**
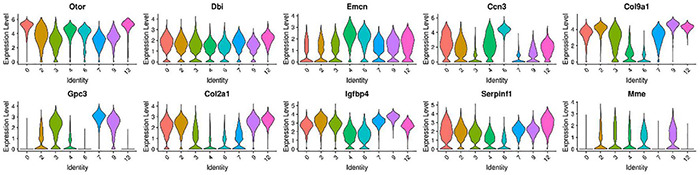
Violin plots showing select genes that are deferentially expressed in the GER cluster 0, 3, 4, and 6 with the number of cells decreased from P1 to P14, and GER cluster 2, 7, 9, and 12 with the number of cells increased from P1 to P7 and degenerated and disappeared at P14 days. The Y-axis, log-normalized transcript counts. These eight clusters had similar expression characteristics.

**FIGURE 4:**
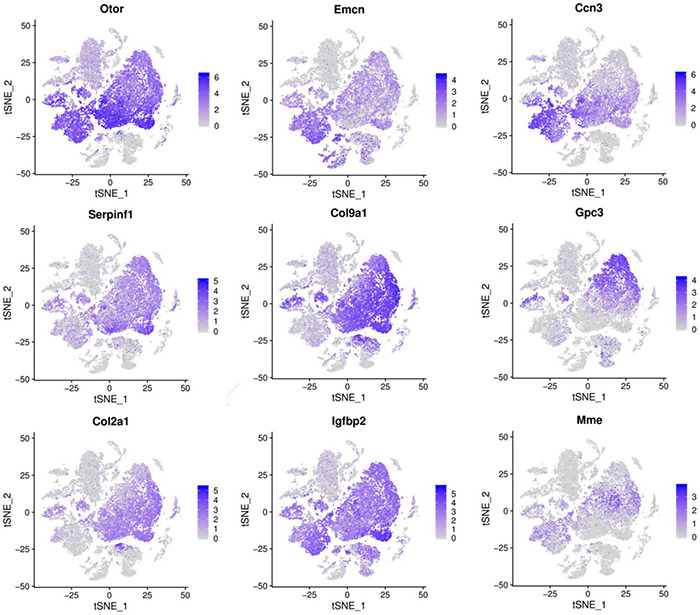
Cells landscape of cochlea duct revealed by scRNA-seq analysis, which was performed on single-cell suspensions pooled from P1, P7, and P14. All samples were analyzed using canonical correlation analysis with the Seurat R package. Cells were clustered using a graph-based shared nearest-neighbor clustering approach plotted by tSNE plot. Feature Plots showing transcript accumulation for specific cell marker genes in individual cells of clusters 0, 2, 3, 4, 6, 7, 9, 12. Color intensity indicates the relative transcript level for the indicated gene in each cell.

We further selected the characteristic genes Otor, Dbi, Emcn, Ccn3, Col9a1, Gpc3, Col2a1, Igfbp4, Serpinf1, and Mme in these eight cell clusters, and analyzed the expression patterns of these genes (Figure 3). It can be seen from the gene expression pattern map that the Otor gene was highly expressed on clusters 0, 2, 4, 6, and 12, with a higher expression on clusters 0, 4, and 12. Dbi, Slc1a3, and Ccn3 genes were consistently expressed on clusters 0, 3, 4, and 6; Gpc3 was highly expressed on clusters 3, 7, and 9, and Col2a1 had similar expression characteristics on clusters 0, 2, 9, and 12. The Igfbp40 gene was present in clusters 2, 9, and 12, while the Serpinf1 gene had similar expression characteristics in clusters 0, 2, 7, 9, and 12 (Figure 4).

Based on the expression levels of genes in different clusters and the spatial regional distribution of gene expression on the t-SEN map, we found that clusters 0, 2, 3, 4, 6, 7, 9, and 12 have similar expression characteristics, among which clusters 4 and 6, clusters 0, 3, and 12, and clusters 2, 7, and 9 were more similar in spatial distribution and gene expression patterns. Through the dynamic changes, gene expression patterns, and spatial distribution of the above cell clusters, we believe that clusters 2, 7, 9, and 12 may be other subclusters of GER cells, and these subsets may have the ability to differentiate or transdifferentiate into other cells based on their proliferative properties from P1 to P 7 (Figure 2).

### scRNA-Seq Identifies Three Inner Hair Cell Subtypes

**FIGURE 5:**
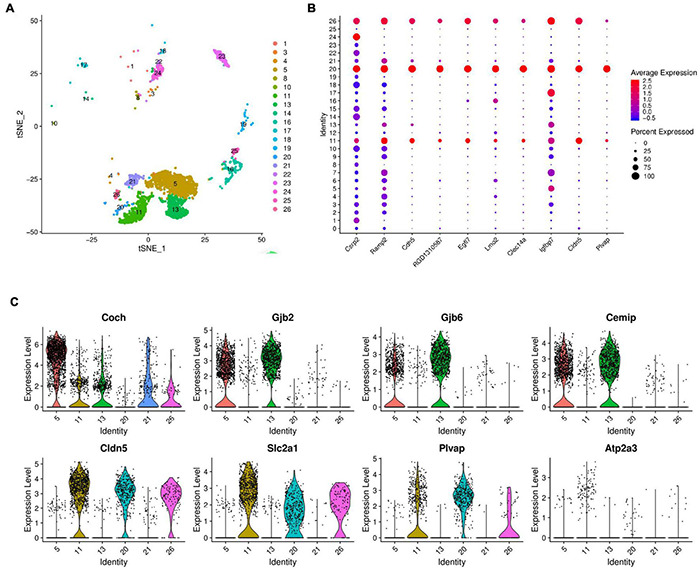
Gene expression in inner cell clusters. **(A)** The t-SEN plots of clusters 11, 20, and 26 were closely linked to clusters 5, 13, and 21 in terms of spatial structure. **(B)** Dot plots representing gene expression levels of different cell clusters. Each dot was sized to represent the proportion of cells of each type expressing the marker gene and colored to represent the mean expression of each marker gene across all cell clusters. **(C)** Violin plots showing select genes that are deferentially expressed in the clusters of 11, 20, 26, and clusters 5, 13, and 21. Y-axis, log-normalized transcript counts.

As seen from the t-SEN plot, cluster 11 was closely linked to clusters 20 and 26 in terms of spatial structure and was significantly increased at P14 as with clusters 5, 13, and 21 (Figure 5A). Analysis of gene expression in these cell clusters revealed that clusters 11, 20, and 26 had very similar gene expression consistency with significant differences from Cluster 5, Cluster 13, and Cluster 21 (Figure 5B). In addition, the spatial distribution of t-SEN shows a segmentation but close spatial location, suggesting a close functional association between these cell clusters.

The violin plot further analyzed the expression of Coch, Gjb2, Gjb6, Cemip, Cldn5, Slc2a1, Atp2a3, Plvap genes in cluster 11 and compared the results with those of clusters 20, 26, 5, 13, and 21. The plot shows that clusters 11, 20, and 26 have similar gene expressions, while clusters 5, 13, and 21 have distinct gene expression characteristics of the supporting cells (Figure 5C).

Three support cell-specific genes, Coch, Gjb2, and Gjb6, are significantly high-expressed in clusters 5 and 13, and Gjb2 and Gjb6 genes encode gap junction proteins (Connexin, Cx) 26 and 30, which constitute the major isoforms of gap junction channel coupling between cochlear support cells (Rabionet et al., 2000; Meşe et al., 2007). Support cells are coupled to each other *via* Cx26 and/or Cx30 gap junction channels or hemichannels, forming a network of support cells that transmit ions, signals, and nutrient molecules and constitute the microenvironment of nearby or surrounding hair cells (Johnson et al., 2017; Mammano, 2019). Both impaired differentiation of supporting cells and impaired substance or signaling between supporting cells can cause impaired auditory development leading to congenital or acquired deafness, as has been demonstrated in transgenic mouse models of deafness (del Castillo et al., 2002). Cochlear hair cell regeneration studies have further confirmed that regenerating hair cells cannot obtain functional maturity without the provision of an appropriate and stable microenvironment by supporting cells, suggesting that these three clusters may play a major regulatory role in the development of cochlear hearing as non-sensory supporting cells (Roccio et al., 2020). In addition, the violin plot showed that Cldn5, Slc2a1, Plvap, Atp2a3 were more highly expressed on clusters 11, 20, and 26. Previous transcriptome studies on mice have shown that Atp2a3, Cabp2, and Shtn1 are specific marker genes for inner hair cells (Pirvola et al., 1995; Liu et al., 2014; Wiwatpanit et al., 2018), and our study found consistent high expression of the Atp2a3 gene specifically on clusters 11, 20, and 26 (Figure 5C), which was consistent with Kolla’s study according to the results of single-cell RNA Sequencing (Kolla et al., 2020). Therefore, we tentatively considered the above three clusters as different subtypes of inner hair cells.

### Inner Hair Cell Trajectory Development

**FIGURE 6:**
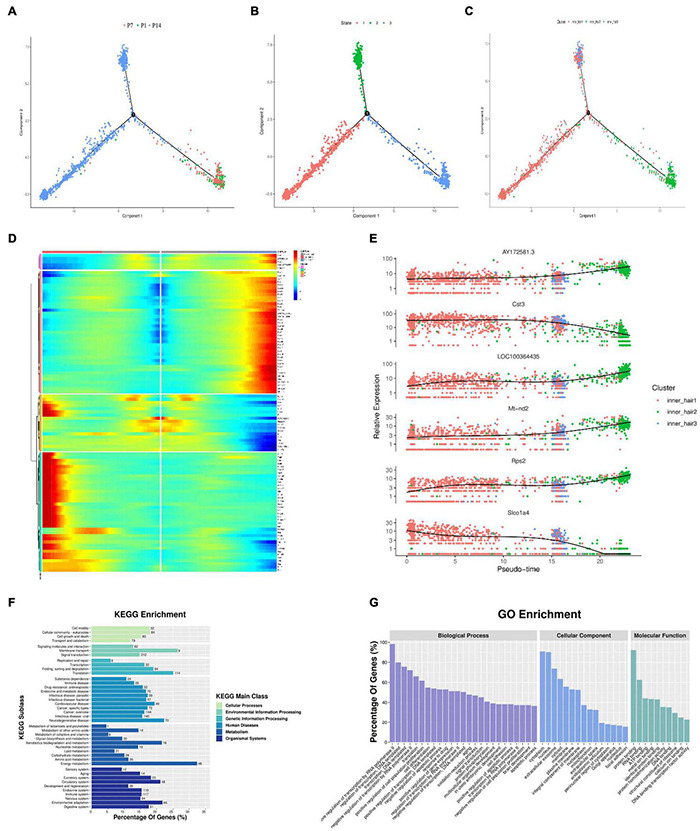
Pseudotime trajectory of the inner hair cell. **(A–C)** Monocle analyses show the development of inner hair cells at different cell developmental periods **(A)**, developmental states **(B)**, and branches of development **(C)**. **(D)** Heatmap of DEGs in different blocks along the pseudotime trajectory. The Red color represents an abundant expression of genes. **(E)** Dynamic gene expression with pseudotime trajectory. **(F)** GO enrichment analysis of genes in cells through fate 1 and fate 2. **(G)** Functional enrichment analyses with KEGG performed with the enriched genes in cells through fate 1 and fate 2.

The developmental trajectories of the three different subtypes of inner hair cells were analyzed, and the results showed the existence of three different cell developmental periods, developmental states, and a branch of development (Figures 6A,B). The cells started to develop from state 1 and transformed into state 2 and state 3 by the first branch.

The main cells in state 1 are cluster 20, with a small number of clusters 11 and 26.

By the time of the first branch, cluster 20 gradually degenerated, and clusters 11 and 26 gradually increased, with cluster 11 increasing more markedly, suggesting that cluster 11 is the main inner hair cell subtype in the maturing cochlear hearing (Figure 6C). As seen from the genetic heat map of cell fate transitions, the genes that were highly expressed during the transition toward Fate1 were mainly Acta2, Tagln, Rgs5, Col4a1, Dcn, GSn. The genes that were highly expressed during the transition toward Fate2 were mainly LOC100364435, Rpsa, Rpl17, Rack1, Otir, Actg1 (Figure 6D).

Throughout the cell developmental trajectory, we found AY172581.3, Cst3, LOC100364435, Mt-nd2, Rps2, Slco1a4 were the major regulatory genes, with the expression of Cst3 and Slco1a4 downregulated and the expression of AY172581.3, LOC100364435, Mt-nd2, Rps2 upregulated (Figure 6E). GO function and KEGG signaling pathway analysis of the genes enriched in Fate1 and Fate 2 cell fate transitions showed that genes were mainly enriched in positive regulative of transcription by RNA polymerase-II of Biological process, the cytoplasm of cellular components, and Protein binding in molecular function of GO functional analysis (Figure 6F and Supplementary Table 2), and KEGG signaling pathway analysis showed genes mainly enriched in the Ribosome signaling pathway (Figure 6G and Supplementary Table 3).

### scRNA-Seq Identifies Two Outer Hair Cell Subtypes

**FIGURE 7:**
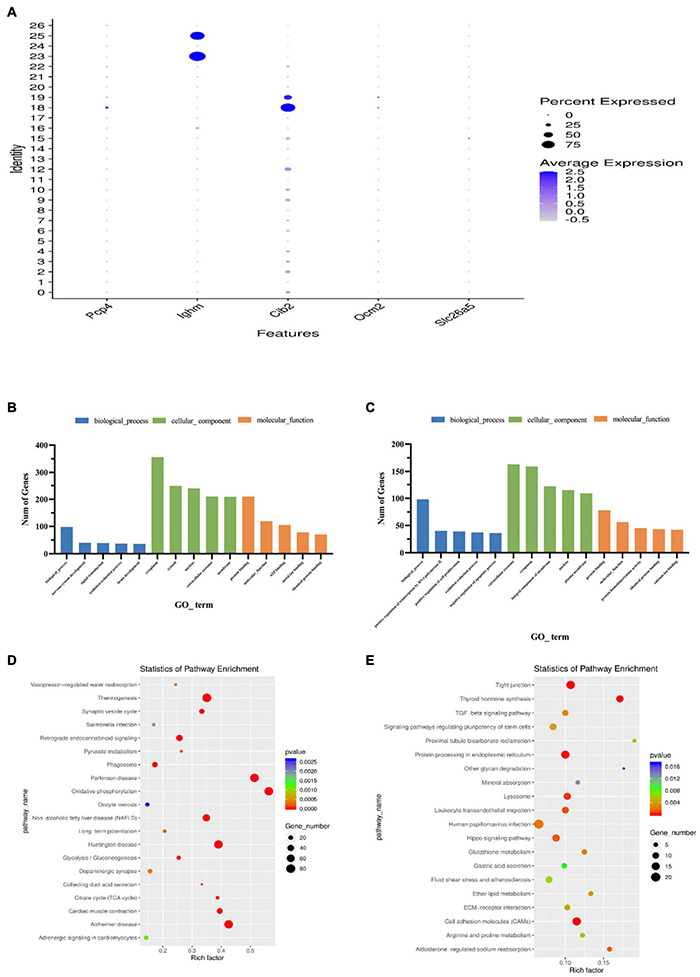
Features of outer hair cell subtypes. **(A)** Accumulation of marker gene transcripts by cluster. This dot plot indicates the level of marker gene expression (dot intensity) and the fraction of cells in each cluster expressing a given marker gene (dot size). **(B)** GO enrichment analysis of genes for cluster 18. **(C)** GO enrichment analysis of genes for cluster 19. **(D)** Functional enrichment analyses using KEGG pathways for cluster 18. **(E)** Functional enrichment analyses using KEGG pathways for cluster 19. The x-axis is the item of go function, the ordinate represents the enriched genes by each item, and the cycle size indicates the significance and corresponding significance values displayed as log10 (*P*-value).

Single-cell sequencing results showed that Cib2 was found to be significantly differentially expressed in clusters 18 and 19, with cluster 18 being more highly expressed (Figure 7A). The Cib2 protein is responsible for maintaining Ca^2+^ homeostasis in cells and interacting with integrins-transmembrane receptors essential for cell adhesion, migration, and activation of signaling pathways, and is widely expressed in various human and animal tissues, mainly in skeletal muscle, nervous tissue, inner ear, and retina (Jacoszek et al., 2017). Now, Cib2 has been added to the extensive list of genes associated with hearing, loss, and previous studies have been shown that Cib2 is a specific gene expressed on outer hair cells (Riazuddin et al., 2012; Burns et al., 2015). Single-cell transcriptome sequencing based on P7-day mice by Kolla et al. (2020) also showed that Cib2 was highly and nearly specific expressed in outer hair cells clusters (White et al., 2006). In this study, gene expression analysis revealed that Cib2 was significantly higher and specifically expressed on both clusters 18 and 19, which were tentatively considered as two different subtypes of outer hair cells. GO functional analysis showed that both clusters 18 and 19 are enriched in the biological process. In terms of cellular components, cluster 18 is mainly enriched in the cytoplasm, and cluster 19 is mainly enriched in the Extracellular exosome. Both clusters are enriched in Protein binding in molecular function (Figures 7B,C and Supplementary Tables 4, 5). KEGG signaling pathway analysis revealed that Cluster18 was mainly enriched in the Thermogenesis signaling pathway, while Cluster19 was mainly enriched in the Tight junction and Cell adhesion molecules signaling pathways (Figures 7D,E and Supplementary Tables 6, 7).

### Developmental Trajectory Analysis Showed Four Greater Epithelial Ridge Cell Subtypes Had the Potential to Transdifferentiate Into Inner and Outer Hair Cells

**FIGURE 8:**
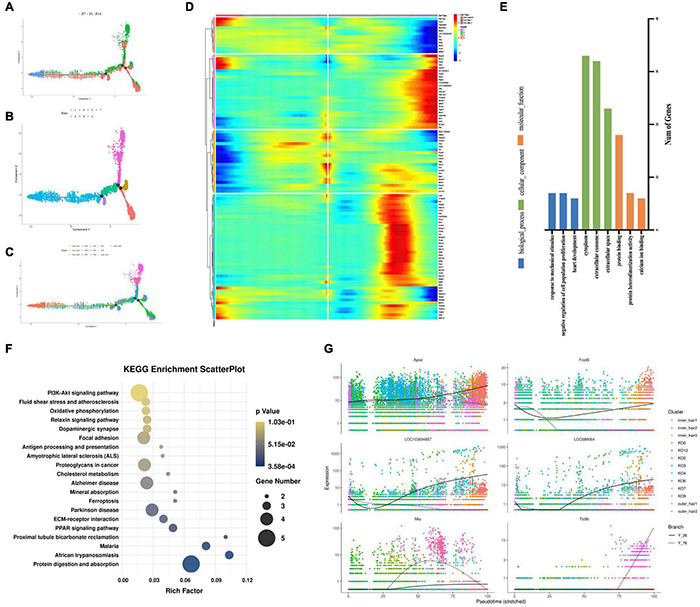
Developmental trajectories of KO cells and inner and outer hair cells. **(A–C)** Monocle analyses show the development of KO cells and inner and outer hair cells. **(D)** Heatmap of different blocks of DEGs along the pseudotime trajectory. **(E)** GO enrichment analysis of genes in Fate 1 and Fate 2 cell fate transitions for KO cells and inner and outer hair cells. **(F)** Functional enrichment analyses using KEGG pathways in Fate1 and Fate 2 cell fate transitions. **(G)** Dynamic gene expression with pseudotime trajectory.

Although cochlear hair cell regeneration does not occur in adult mammals, the neonatal mouse cochlea inner the first two neonatal weeks shows limited and organoid-generating regenerative potential (Oshima et al., 2007). To determine whether GER (KO) cells may have the potential to transdifferentiate into inner and outer hair cells, we further performed the cell development perspective by single-cell RNA sequencing trajectory analysis and found it interesting that the main cell clusters at the beginning of cell development were KO2, KO7, KO9, and KO12, which were firstly increased in the number of cells from P1 to P7, while degenerated and disappeared at P14 days. At the second branch, part of the cells developed toward outer hair cells (Figures 8A–C) and part toward inner hair cells (Figures 8A–C). As seen from the genetic heat map of the cell fate transition, the genes that are highly expressed when the cells are transitioning toward fate 1 are mainly Aldh1a2, Ccn3, Ank2, Stmn1, Map1a, Kif5c. The genes that are highly expressed when the cells are transitioning toward fate 2 are mainly Rps28, Apoe, Ac134224.2, Ptgds, Cst3, Igfbp7, Hbb, Hba-a2, Tagln2, Tmsb4x, Crsp2 (Figure 8D). Go function and KEGG signaling pathway analysis of the genes enriched in Fate1 and Fate 2 cell fate transitions showed that genes were mainly enriched in negative regulation of cell population proliferation of biological process, and cytoplasm in a cellular component, and Protein binding in molecular function (Figure 8E), and KEGG signaling pathway analysis showed genes mainly enriched in the PI3K-AKT signaling pathway (Figure 8F). In the whole process of cell development, Apoe, Fxyd5, LOC103694857, LOC689064, Mia, Itc9b are the main regulatory genes and are upregulated (Figure 8G).

### Gene Ontology Function and Kyoto Encyclopedia of Genes and Genomes Pathway Analysis the Four *Trans*-Differentiations Potential Greater Epithelial Ridge Cell Clusters

**FIGURE 9:**
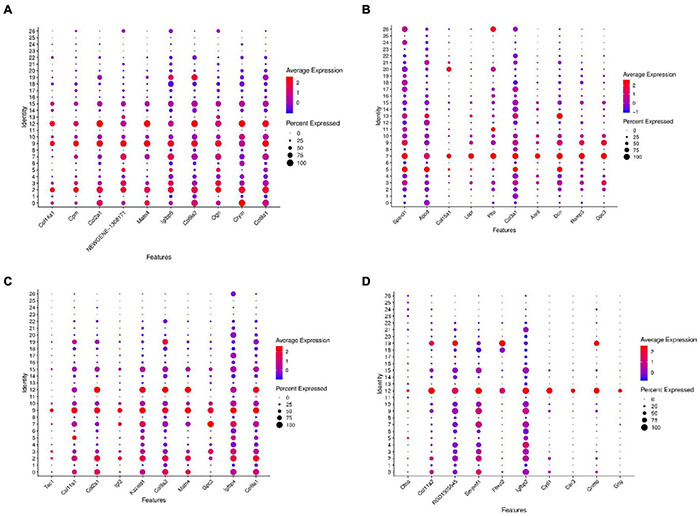
Dot plots representing expression levels of the KO2, KO7, KO9, and KO12 clusters. All samples were analyzed using dot plot analysis with the Seurat R package. Expression levels of the top ten genes on cluster 2 **(A)**, cluster 7 **(B)**, cluster 9 **(C)**, cluster 12 **(D)** are shown. Each dot was sized to represent the proportion of cells of each type expressing the marker gene and colored to represent the mean expression of each marker gene across all cells.

Further gene expression analysis of the above four clusters with *trans*-differentiation potential revealed that they all have similar gene expression patterns. Relatively specific highly expressed genes were Crym, Col9a2, Col2a1, Col9a1 in cluster 2 (Figure 9A), Lepr, Ramp3, Gpc3 in cluster 7 (Figure 9B), Igfbp4, Gpc3 in cluster 9 (Figure 9C), and Cytl1, Cnmd, Serpinf1, and Col11a2 in cluster 12 (Figure 9D).

**FIGURE 10:**
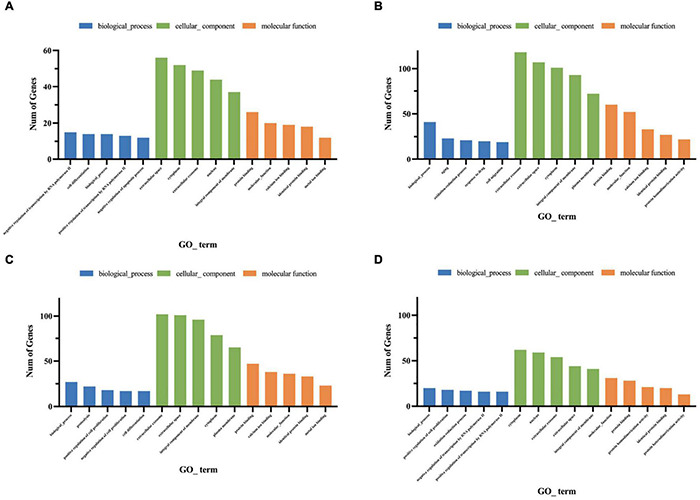
Gene ontology enrichment analysis of genes for cluster 2 **(A)**, cluster 7 **(B)**, cluster 9 **(C)**, cluster 12 **(D)**. The Go functions include molecular functions, cellular components, and biological processes. The x-axis is the item of go function, and the ordinate represents the enriched genes by each item.

**FIGURE 11:**
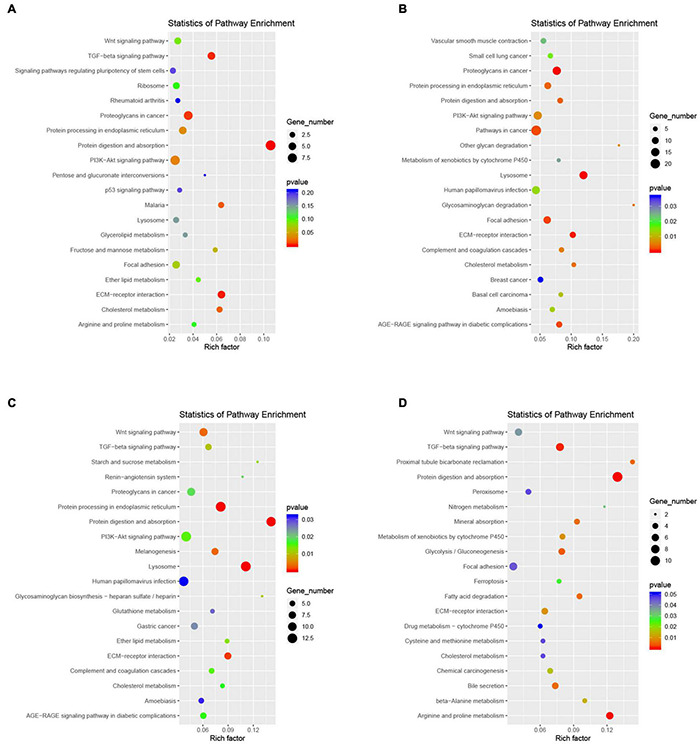
Functional enrichment analyses using KEGG pathways for cluster 2 **(A)**, cluster 7 **(B)**, cluster 9 **(C)**, cluster 12 **(D)**. The cycle size indicates the corresponding significance values displayed as log10 (*P*-value). The much bigger the triangle size, the much more genes enriched in this pathway.

Gene ontology functional analysis showed that all four cell clusters were enriched in Protein binding in terms of molecular function, cluster 2 was mainly enriched in Extracellular space (Figure 10A and Supplementary Table 8), clusters 7 and 9 were mainly enriched in Extracellular exosome (Figures 10B,C and Supplementary Tables 9, 10), and cluster12 was mainly enriched in Cytoplasm (Figure 10D and Supplementary Table 11). Cluster 2 is mainly enriched in the Negative regulation of transcription by RNA polymerase II (Figure 10A), while the other three cell groups are mainly enriched in the biological process (Figures 10B–D). KEGG signaling pathway analysis revealed the following enriched signaling pathways: Cluster 2 (Figure 11A) and cluster 12 (Figure 11D) were mainly in Protein digestion and absorption; Cluster 7 was mainly in the pathway in cancer (Figure 11B); Cluster 9 was in Protein digestion and absorption, in addition to two signaling pathways, Protein processing endoplasmic reticulum and Lysosome (Figure 11C).

### Complex Cell-Cell Communication Networks Exist in Greater Epithelial Ridge (Kölliker’s Organ) Cells and Cochlear Inner and Outer Hair Cells

**FIGURE 12:**
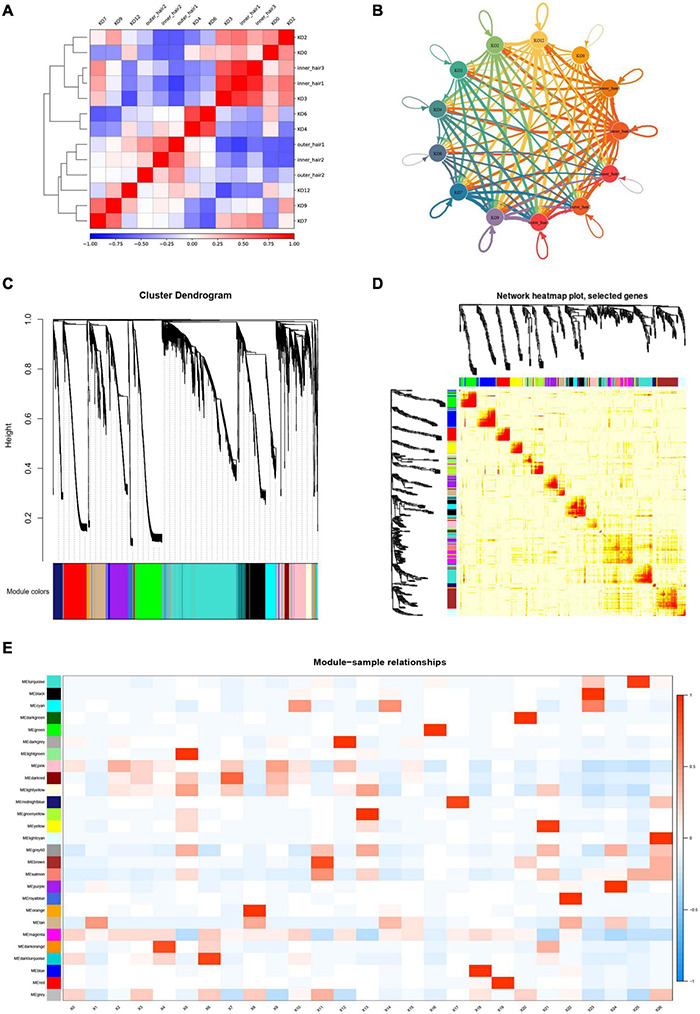
Cell-cell communication networks and WGCNA analysis of KO cell clusters and hair cell clusters. **(A)** Heatmap in KOs and hair cells. The red color indicates a significant interaction. **(B)** Capacity for intercellular communication among KOs and hair cells. Each line color indicates the ligands expressed by the cell population represented in the same color (labeled). The lines connected to cell types expressed the cognate receptors. The line thickness was proportional to the number of ligands when cognate receptors were present in the recipient cell type. **(C)** The dendrogram of gene modules built by WGCNA. **(D)** Network heatmap plot. The module corresponds to branches. The bright red color of nodes represented stronger the genetic correlation of this module. **(E)** Module-trait relationship between different gene modules. The number in each cell represents the degree of correlation, and red means positive correlation at this stage; blue means negative correlation at this stage. Different colors represent the diverse specific gene modules detected by WGCNA.

To systematically assess the associated complex cellular responses, we attempted to map ligand-receptor interactions with our scRNA-seq data to better understand cellular behaviors and responses to neighboring cells in the cochlear basal membrane. We considered the expression levels of ligands and receptors within GER cell clusters and the inner and outer hair cell clusters and predicted molecular interactions between cell populations *via* specific protein complexes. We then generated a potential intercellular communication network among all cells in the GER cell clusters and hair cell clusters separately. Broadcast ligands for which cognate receptors were detected and manifested broad communication between GER cell and the inner and outer hair cell (Figures 12A,B and Supplementary Figures 1–13). The WGCNA method was further used to analyze the gene association patterns between different subtypes of GER cells and the inner and outer hair cells of the cochlea (Figures 12C,D). We found that the blue module was the most significant and associated with the GV phase, while the yellow module was the most significant and associated with the MI phase and the magenta module was the most significant and associated with the MII phase (Figure 12E).

### Fluorescence *in situ* Hybridization Shows the Presence of Dynamic Gene Expression Changes in the Cochlear Basal Membrane

**FIGURE 13:**
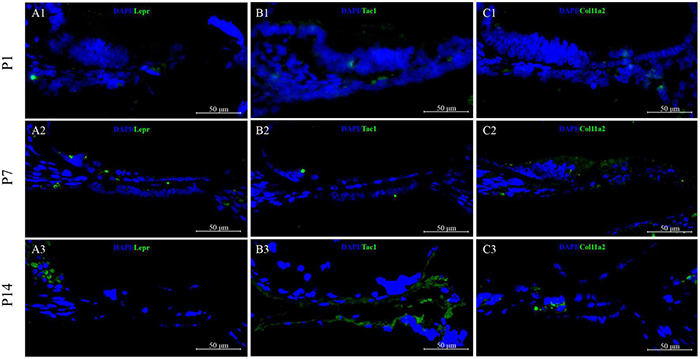
Validation of high expression gene of GER cell clusters at P1, P7, and P14. Lepr was low expressed in the GER region at P1 **(A1)**, and upregulated expression in GER and inner hair cell region at P7 **(A2)**, but the expression was significantly reduced and almost disappeared at P14 **(A3)**. Tac1and Col11a2 were low expressed at P1 **(B1,C1)** but showed a significantly upregulated expression in GER at P7 **(B2,C2)**. While during the P14 period, Tac1and Col11a2 were nearly disappeared in the GER region and were centrally expressed in the hair cell region **(B3,C3)**.

To validate the cell-type-specific genes, we used fluorescence *in situ* hybridization (FISH) to localize transcripts in cross-sections from P1 to P14 cochlear basal membrane (Figure 13). Three genes with high expression on clusters 7, 9, and 12 based on scRNA-seq results were selected for FISH: Lepr, Tac1, and Col11a2. The FISH results showed that Lepr was low expressed in the GER region at P1 (Figure 13A1), and sign up the regulated expression in GER and inner hair cell region at P7 (Figure 13A2), but almost disappeared in GER and inner hair cell region at P14 (Figure 13A3). While we found Tac1 and Col11a2 genes showed patterns of expression that were consistent with the single-cell results. From the FISH results, it could be seen that Tac1and Col11a2 were low expressed to the whole cochlear basal membrane at P1 (Figures 13B1,C1), and up the regulated expression in GER at P7 (Figures 13B2,C2). While at P14, Tac1, and Col11a2 were nearly disappeared in the GER region and were centrally expressed in the hair cell region (Figures 13B3,C3).

## Discussion

Greater epithelial ridge is a cluster of columnar cells located on the medial side of the cochlear hair cells, which is temporarily present during the development of the cochlea and is one of the signs of immaturity (Hinojosa, 1977). During the development of hearing, the GER gradually degenerates from the basal turn to the apex turn. After the cochlea matures and hearing appears, the GER degenerates and is replaced by cells from the internal sulcus (Lim and Anniko, 1985). Single-cell sequencing studies by Kolla et al. (2020) identified four distinct GER (KO) cell subtypes (KO1, KO2, KO3, and KO4) in the cochlear GER of P1-day CD1 female mice. These four cell subtypes have massive highly expressed genes, including high expression of Dcn, Ddost, Pdia6, Rcn3, Sdf2l1 in KO1 cell; high expression of Cpxm2, Ctgf, Fkbp9, Kazald1, Tectb in KO2 cell; Cst3, Gjb6, Net1, Tectb, Tsen15 in KO3 cell; high expression of Calb1, Crabp1, Epyc, Itm2a, Stmn2 in KO4 cell. The study defined the cell subtypes with high expression of Calb1 gene in the medial region of GER as the medial region of GER cell subtype based on the expression of Calb1 and Fabp7 and the outer region of GER. The other three subtypes in the outer region of GER with high expression of Fabp7 gene were further defined as L.KO1, L.KO2, and L.KO3 cells, respectively. Kolla et al. (2020) showed that these four cell subtypes are highly similar in terms of gene expression patterns and metabolic functions, but at the same time they are heterogeneous, and it is tentatively considered that these four cell subtypes are different subtypes of GER cells.

Considering that the cochlear auditory development of P7 rats is not fully mature, it is still in a process of orderly differentiation and gradual developmental maturation (Tritsch et al., 2007; Tritsch and Bergles, 2010). Based on our previous study (Chen et al., 2021), we further selected the P14 time point jointly with the three key periods of P1, P7 for a single-cell transcriptomic study, and we found that the four previously identified subtypes of GER cell populations 0, 3, 4, and 6 gradually decreased from P1 to P14, and disappeared by P14 days (Figure 1B). However, it is interesting and noteworthy that we found four cell clusters (clusters 2, 7, 9, and 12) with similar spatial distribution and gene expression (Figures 2, 3). These four cell clusters gradually increased in the number of cells from P1 to P7, but gradually decreased in number from P7 to P14 days, and disappeared by P14 days, which seems to be consistent with the outcome of the disappearance of GER cells. In terms of gene expression patterns, these four cell clusters are highly expressed in Col2a1, Col9a1, Col9a2, and Col11a2, which are the major gene members of the Col family, and mutations in these genes are associated with hearing loss (Richards et al., 2013; Hofrichter et al., 2019; Kjellström et al., 2021).

In addition, based on the KEGG signaling pathway showed that these clusters are mainly enriched in the Protein digestion and absorption signaling pathway, which was previously shown to regulate intracellular Ca^2+^ concentration and dynamic homeostasis, inducing cellular processes such as cell migration proliferation, and differentiation. GER supports the spontaneous cellular release of adenosine triphosphate (ATP), which acts as a paracrine receptor on the P2X purinergic receptors of neighboring IHCs to produce phospholipase C (PLC)-dependent inositol triphosphate (IP3), the release of Ca^2+^ from the intracellular calcium pool, and the release of the neurotransmitter glutamate from IHCs, which activates type I spiral neurons (SGNs) to generate action potentials, thereby mimicking the mechanical-electrical signal transduction effect triggered by sound waves transmitted through the external ear canal, allowing IHCs spontaneously increase the frequency of action potential delivery and promote the functional maturation of IHCs (Tritsch et al., 2007; Majumder et al., 2010; Rodriguez et al., 2012; Mammano, 2013). We consider that these four cell clusters may be other subtypes of GER cells and regulate the balance of Ca^2+^ through the mechanisms described above, which in turn induce and promote the maturation or possible *trans*-differentiation of cochlear hair cells into inner and outer hair cells.

Adult mammalian cochlear hair cells have no regenerative capacity, but neonatal mouse cochlear hair cells show a limited and transient regenerative potential, and studies have shown that this regenerative potential is largely attributable to the non-sensory cells in the neonatal mouse cochlea (Shou et al., 2003; Woods et al., 2004; Gubbels et al., 2008). The mitotic division of neonatal murine cochlear non-sensory cells is normally quiescent, and the regenerative capacity of these cells is further activated during early apoptosis of hair cells due to various factors (Shi et al., 2013; Bramhall et al., 2014; Cox et al., 2014; Hu et al., 2016). GER cells are a transient population of newborn cells with the ability to regenerate and transform with some cell subtypes located within the GER region that retain the characteristics of precursor sensory cells (Zheng and Gao, 2000; Kelly and Chen, 2007; Driver et al., 2008). GER cells maintain a high degree of morphological consistency, and Kolla et al. (2020) found the existence of two types of precursor sensory cells (pro-sensory cells) expressing Cdkn1b and Sox2 marker genes based on single-cell sequencing technology in E14 and E16-day mouse cochlea, which are located in the medial precursor sensory cell population (MPsCs) and lateral precursor sensory cell population (LPsCs) of GER structures, respectively, while the results of single-cell trajectory analysis showed that MPsCs cells have a clear ability to convert to IPhCs or IHCs. LPsCs cells may convert to DCs or OHCs, without clear divergence (Kolla et al., 2020). In addition, Kubota et al. (2021) similarly confirmed that GER cells have a great potential for organoid formation and classified GER cells into three large subtypes S2, S3, and S4 cell subtypes based on single-cell sequencing results. The researchers similarly found in P2 day mice that GER cell subtypes located in the lateral and medial regions could regenerate into hair cells and supporting cells. GER cells cultured at higher densities in the presence of EFI_CVPM [small molecules (CHIR99021, valproic acid, 2-phospho-L-ascorbic acid, and TGFß receptor inhibitor 616452)] were found to be the most efficient cochlear organoid-forming cell population (McLean et al., 2017), with the most lateral GER cell population expressing Lgr5 positivity and the more medially located GER cell population expressing Lgr5 negativity, both having the ability to generate cochlear organoids. Previous studies on the development of other organoids have shown that activation of the WNT signaling pathway can induce high Lgr5 expression and further promote the developmental process (Chai et al., 2011, 2012; Shi et al., 2012, 2013). Therefore, Lgr5 expression in GER cells is not an indicator of stem cells or proliferative potential. The present single-cell transcriptome results also showed that the above-mentioned cell subtypes with proliferative capacity located in the outer part of GER, but its Lgr5 gene expression is not significant, which also suggests that cochlear development is different from other tissues and organs development.

In the present study, our single-cell transcriptomics-based study showed a significant increase in the number of cell clusters 2, 7, 9, and 12 at P7, with more significant increases in cell clusters 7 and 9, only to disappear further at P14. In general, GER cells gradually degenerate and disappear, while other types of supporting cells gradually increase and eventually replace GER cells as the main supporting cells in the cochlea. We consider that clusters 2, 7, 9, and 12 may be subtypes of GER cells with regenerative differentiation potential, which have similar gene expression patterns and biological characteristics, with cluster 7 and cluster 9 being more similar and cluster 2 and cluster 12 being closer. In a rough classification, they can be divided into two large subtypes, but in terms of some specific gene expression, such as Tac1 which is more abundantly expressed on cluster 9, they can be finely divided into four subtypes. To further explore whether these four clusters with proliferative capacity have the potential to transdifferentiate into inner and outer hair cells, we further constructed the developmental trajectory and fate transduction using single-cell pseudo-temporal analysis. The single-cell developmental trajectory shows that the major cell subtypes at the beginning of cell development are clusters 2, 7, 9, and 12 (see Figures 8A–C). As the cells develop further, the four-cell clusters mentioned above gradually develop in two different trajectory directions, with one part developing toward the outer hair cells and the other part shifting toward the inner hair cells. The cell developmental trajectory analysis suggested that these four cell subtypes have the potential to transdifferentiate into outer and inner hair cells, among which cluster 2 and cluster 12 may have the potential to transdifferentiate into outer hair cells (see Figure 8C), while cluster 7 and cluster 9 may only have the potential to transdifferentiate into inner hair cells (see Figure 8C). Since the present study was mainly based on raw letter data, further experimental confirmation is still needed for subsequent studies.

Although studies have shown that GER cells have the potential for organoid regeneration and development, do they have the potential to transdifferentiate into hair cells? Kubota et al. (2021)’s study found that large organoids derived from GER cells contained cells positive for hair cell markers, suggesting that GER cells have the characteristics of precursor sensory cells after mitosis. In addition, it was also found that GER cells can also generate hair cell-like cells after Atoh1 expression (Woods et al., 2004). These findings and the evidence provided by our study suggest that GER cells are a distinct population of cochlear cells characterized by a response to various reprogramming strategies, including phenotypic transformation (*via* Atoh1 expression) and proliferation/de-differentiation, culminating in degeneration and disappearance after maturation of cochlear hearing development.

In conclusion, our study lays the groundwork for elucidating the mechanisms of the key regulatory genes and signaling pathways in the *trans*-differentiation of GER cell subtypes into hair cells and provides potential clues to understand hair cell regeneration and further study of hair cell regeneration. Also, our study reveals the key molecular mechanisms of large epithelial crest supporting cells in promoting cochlear hearing development, which is very important to further elucidate the development of the peripheral auditory system.

## Data Availability Statement

The datasets presented in this study can be found in online repositories. The names of the repository/repositories and accession number(s) can be found below: https://www.ncbi.nlm.nih.gov/geo/query/acc.cgi?acc=GSE195702.

## Ethics Statement

The animal study was reviewed and approved by The Institutional Animal Care and Use Committee of the Shanghai Jiao Tong University School of Medicine.

## Author Contributions

JiC, LS, and JY designed this study. JiC and JuC took the cochlear tissue. SH, BH, YL, SL, FZ, and XS assisted with data acquisition, analysis, and processing. JiC and DG analyzed and interpreted the data and drafted the manuscript. JY, YJ, and LS revised the manuscript. All authors contributed to the article and approved the submitted version.

## Conflict of Interest

The authors declare that the research was conducted in the absence of any commercial or financial relationships that could be construed as a potential conflict of interest.

## Publisher’s Note

All claims expressed in this article are solely those of the authors and do not necessarily represent those of their affiliated organizations, or those of the publisher, the editors and the reviewers. Any product that may be evaluated in this article, or claim that may be made by its manufacturer, is not guaranteed or endorsed by the publisher.

## Footnotes

1 https://support.10xgenomics.com/single-cell-gene-expression/software/pipelines/latest/what-is-cell-ranger, version 3.1.0.

2 http://cole-trapnell-lab.github.io/monocle-release/docs/, version 2.8.0.

3
https://doi.org/10.1201/9781315370279

4 https://doi.org/10.18129/B9.bioc.CellTrails, v1.4.0.

5 https://doi.org/10.18129/B9.bioc.M3Drop, v1.12.0.
