# Supplementary material for: Pseudo-Temporal Analysis of Single-Cell RNA Sequencing Reveals Trans-Differentiation Potential of Greater Epithelial Ridge Cells Into Hair Cells During Postnatal Development of Cochlea in Rats
Source: Front Mol Neurosci. 2022 Mar 16;15:832813. doi: 10.3389/fnmol.2022.832813 (PMC8966675; doi:10.3389/fnmol.2022.832813)
Supplement: Supplementary file 12 [file Table_12.DOCX]

The following statistical programs were used for the data analysis and figure creation.

Filter_Basicinfo.rData（4.79G）

R page：library(monocle)、library(Seurat)

reduceDimension max_components = 2, reduction_method = "DDRTree"。

clustered_spleen_monocle <- newCellDataSet(data,

phenoData = pd,

featureData = fd,

lowerDetectionLimit = 0.5,

expressionFamily = negbinomial.size())

clustered_spleen_monocle <- setOrderingFilter(clustered_spleen_monocle, ordering_genes)

ordering_genes qval < 0.01

clustered_spleen_monocle <- reduceDimension(clustered_spleen_monocle, max_components = 2, reduction_method = "DDRTree")

website: http://cole-trapnell-lab.github.io/monocle-release/docs/

**WGCNA：**

R page：library("WGCNA")

net = blockwiseModules(datExpr, corType = "pearson",

networkType = "unsigned", power = 7, minModuleSize = 30,

mergeCutHeight = 0.25, numericLabels = TRUE, saveTOMs = TRUE,

pamRespectsDendro = FALSE, saveTOMFileBase = mouseTOM)

website: https://mirrors.tuna.tsinghua.edu.cn/CRAN/

**CellphoneDB**

Website：<https://pypi.org/project/CellPhoneDB/>

**Prism version 7.0 (GraphPad Software)**

Serial : GPS-0320559-LFUL-95242

Activation : ACTGP-01337000-00133700-00133700-00133700
