# Supplementary figures and images for: Pseudo-Temporal Analysis of Single-Cell RNA Sequencing Reveals Trans-Differentiation Potential of Greater Epithelial Ridge Cells Into Hair Cells During Postnatal Development of Cochlea in Rats
Source: Front Mol Neurosci. 2022 Mar 16;15:832813. doi: 10.3389/fnmol.2022.832813 (PMC8966675; doi:10.3389/fnmol.2022.832813)

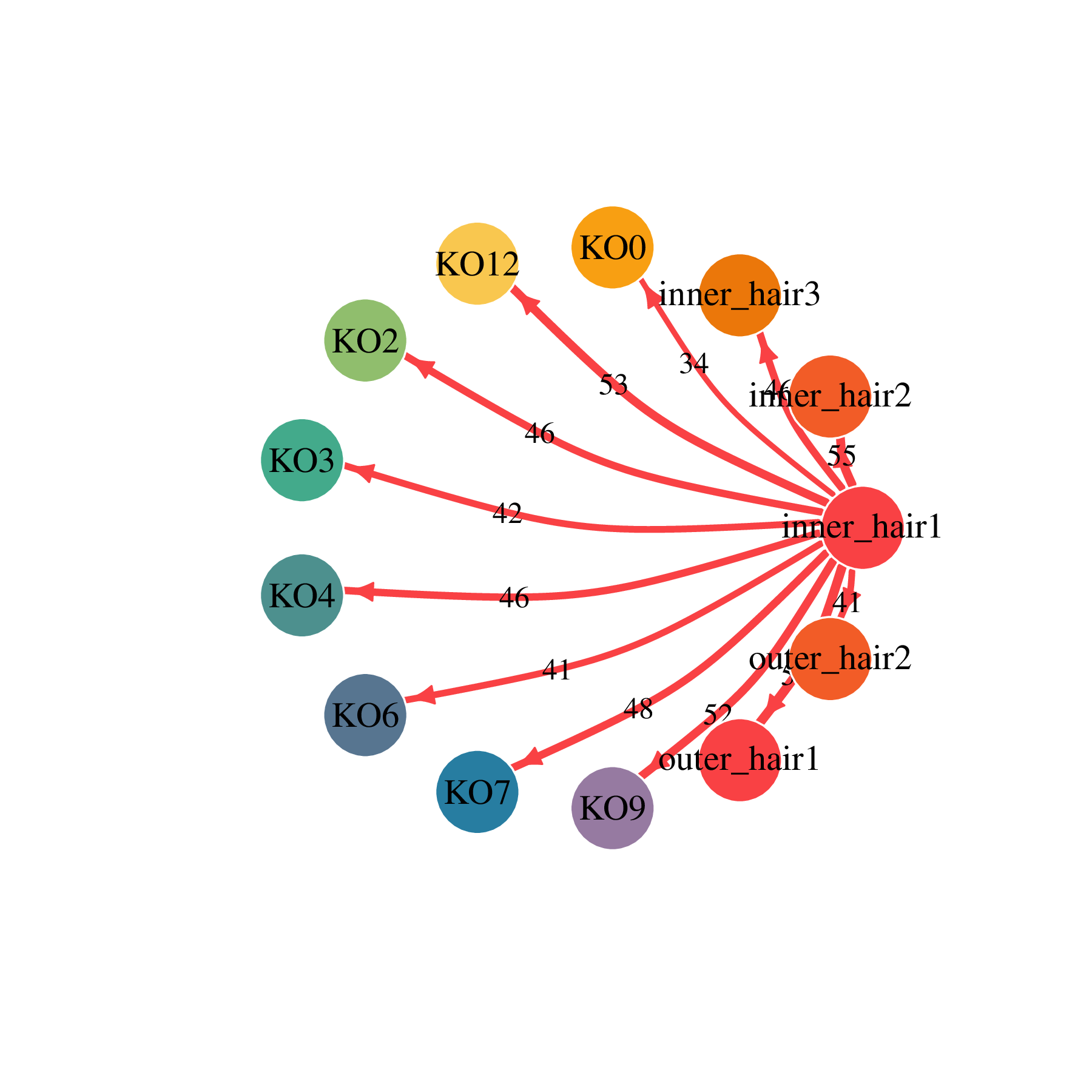

Supplement: Supplementary file 13 [file Image_1.PNG]

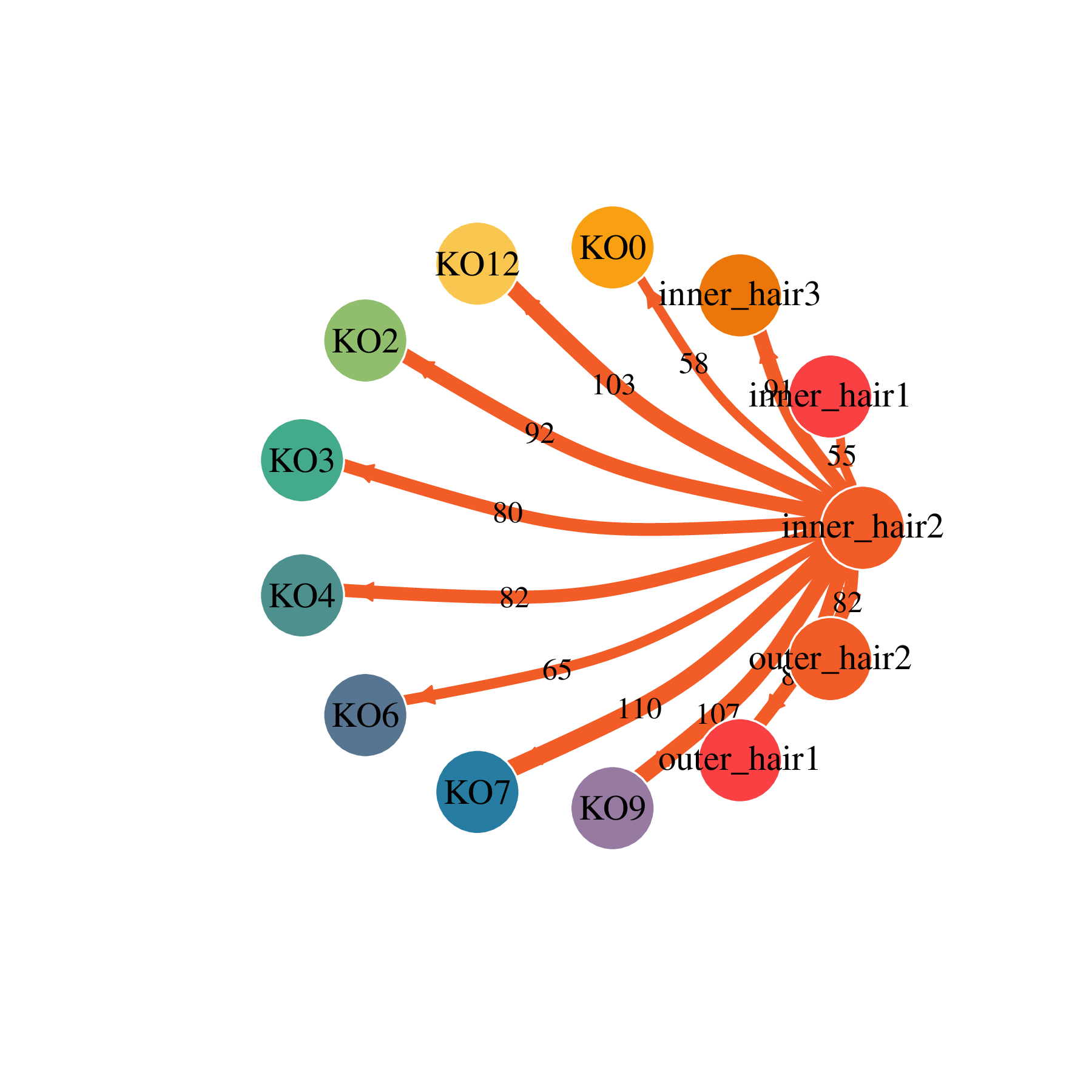

Supplement: Supplementary file 14 [file Image_2.PNG]

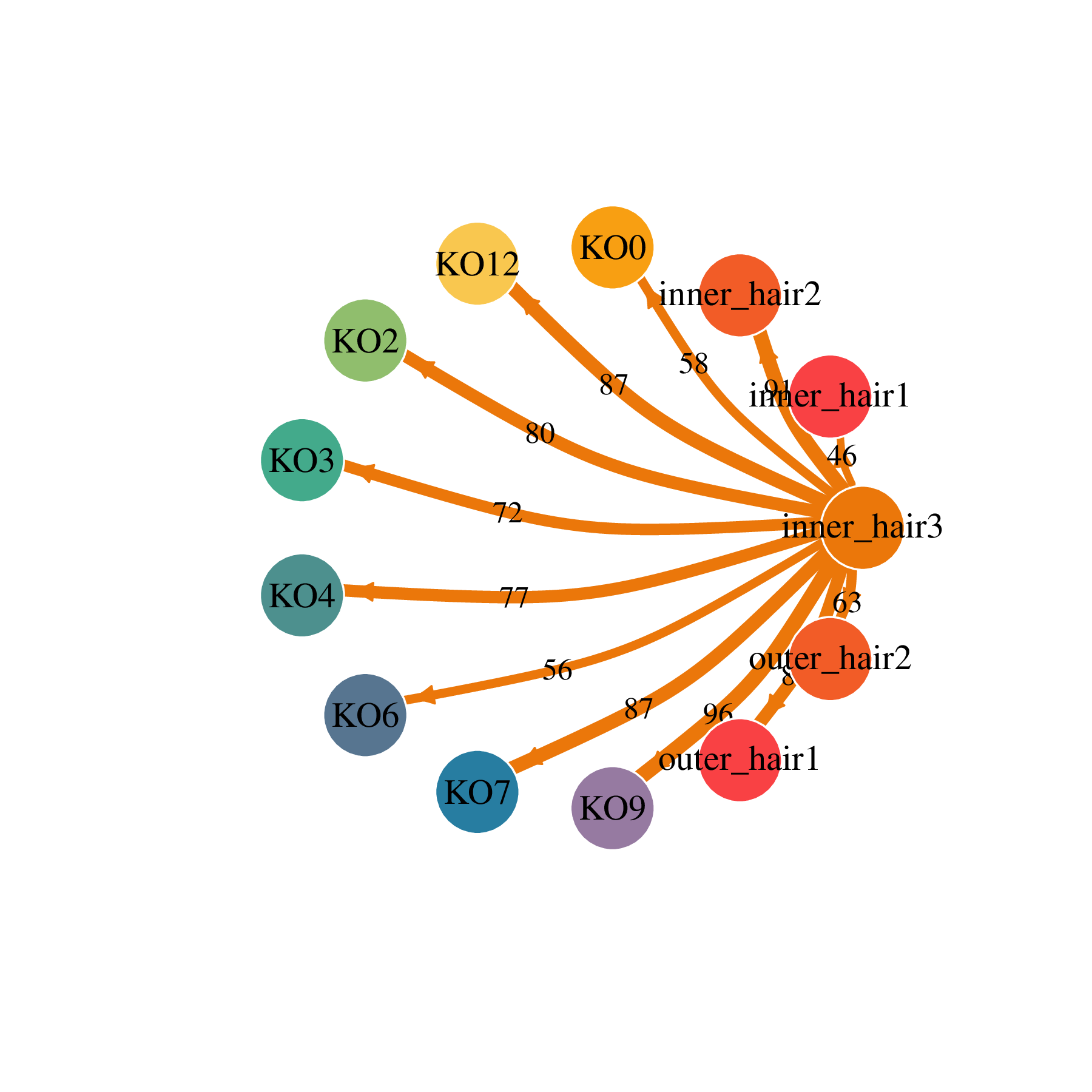

Supplement: Supplementary file 15 [file Image_3.PNG]

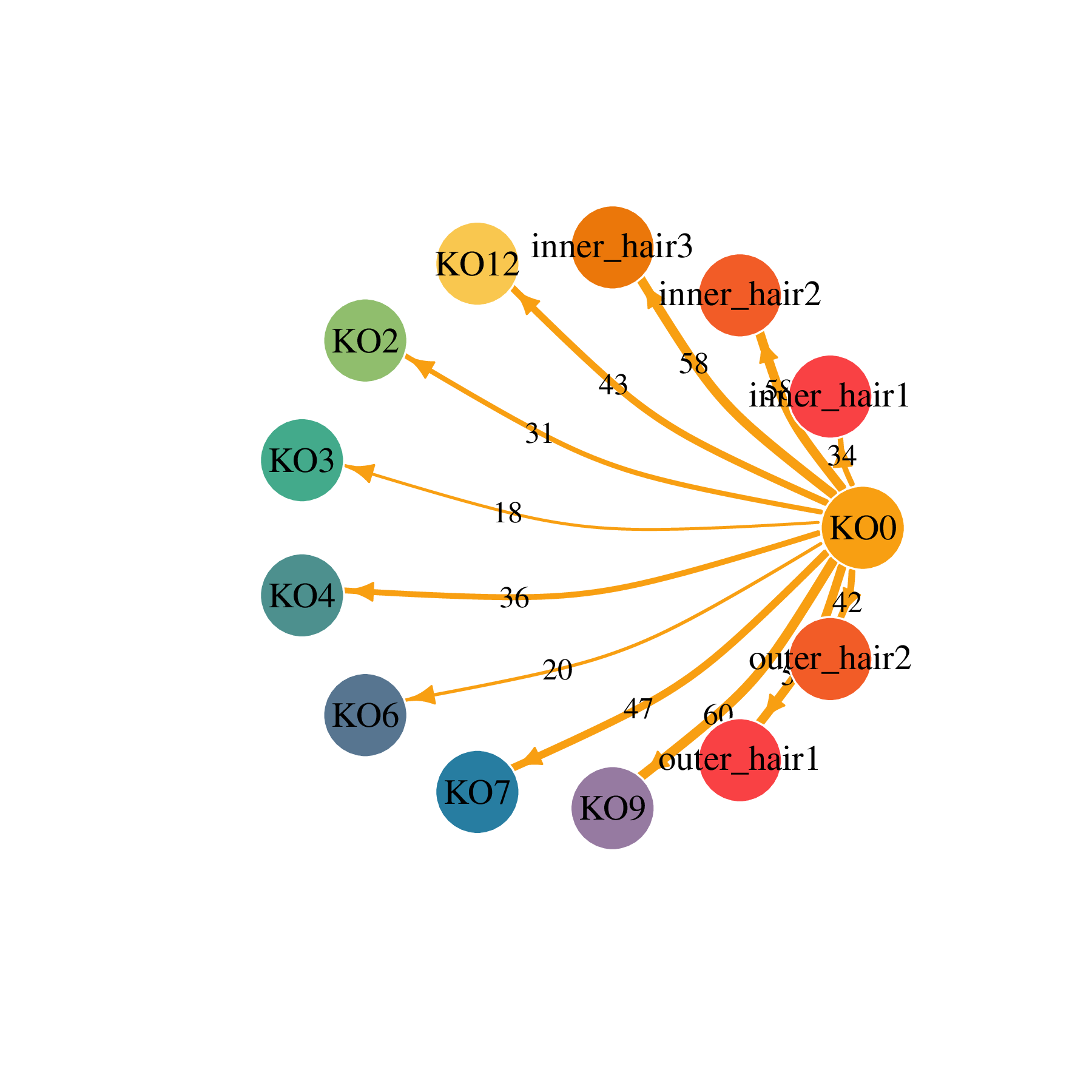

Supplement: Supplementary file 16 [file Image_4.PNG]

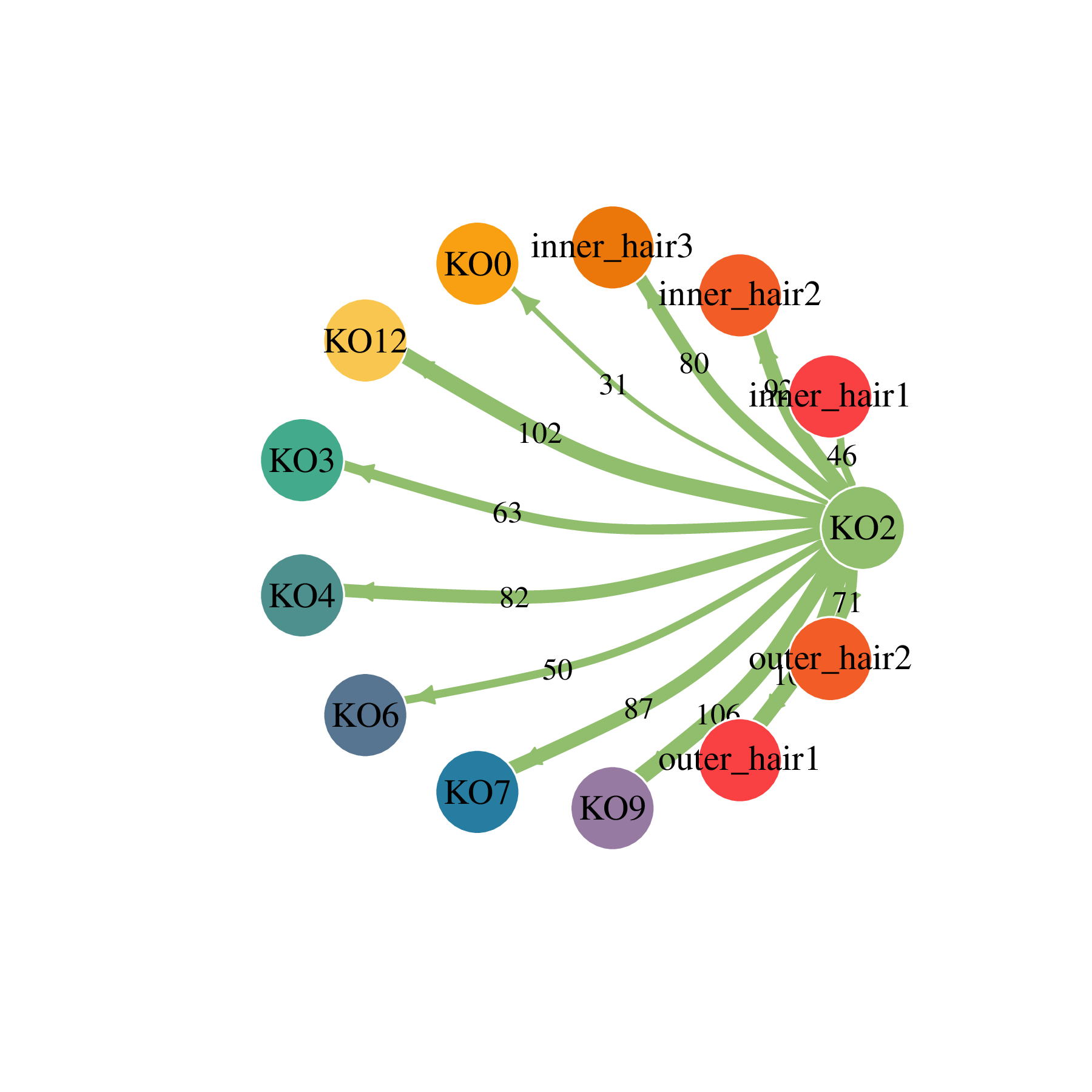

Supplement: Supplementary file 17 [file Image_5.PNG]

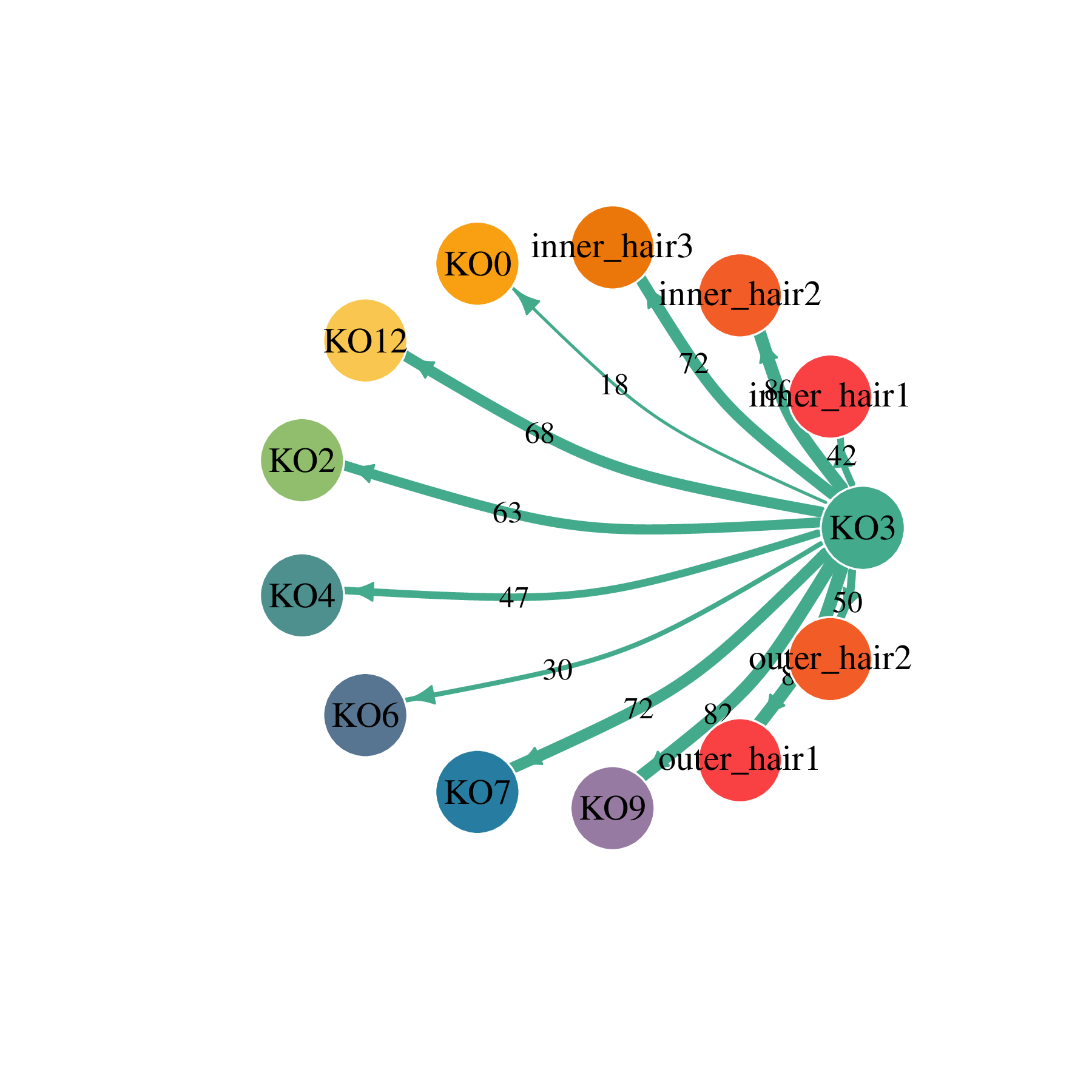

Supplement: Supplementary file 18 [file Image_6.PNG]

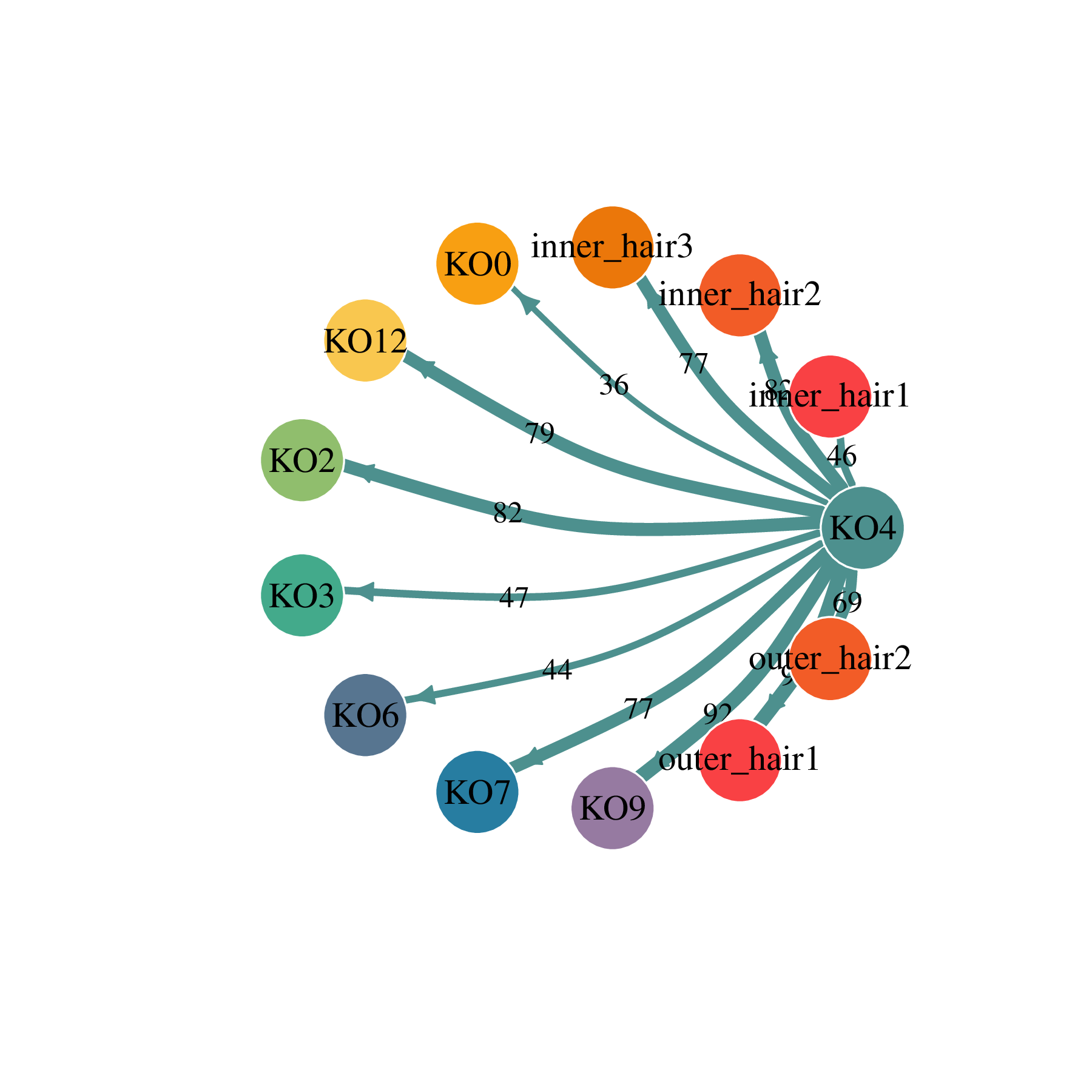

Supplement: Supplementary file 19 [file Image_7.PNG]

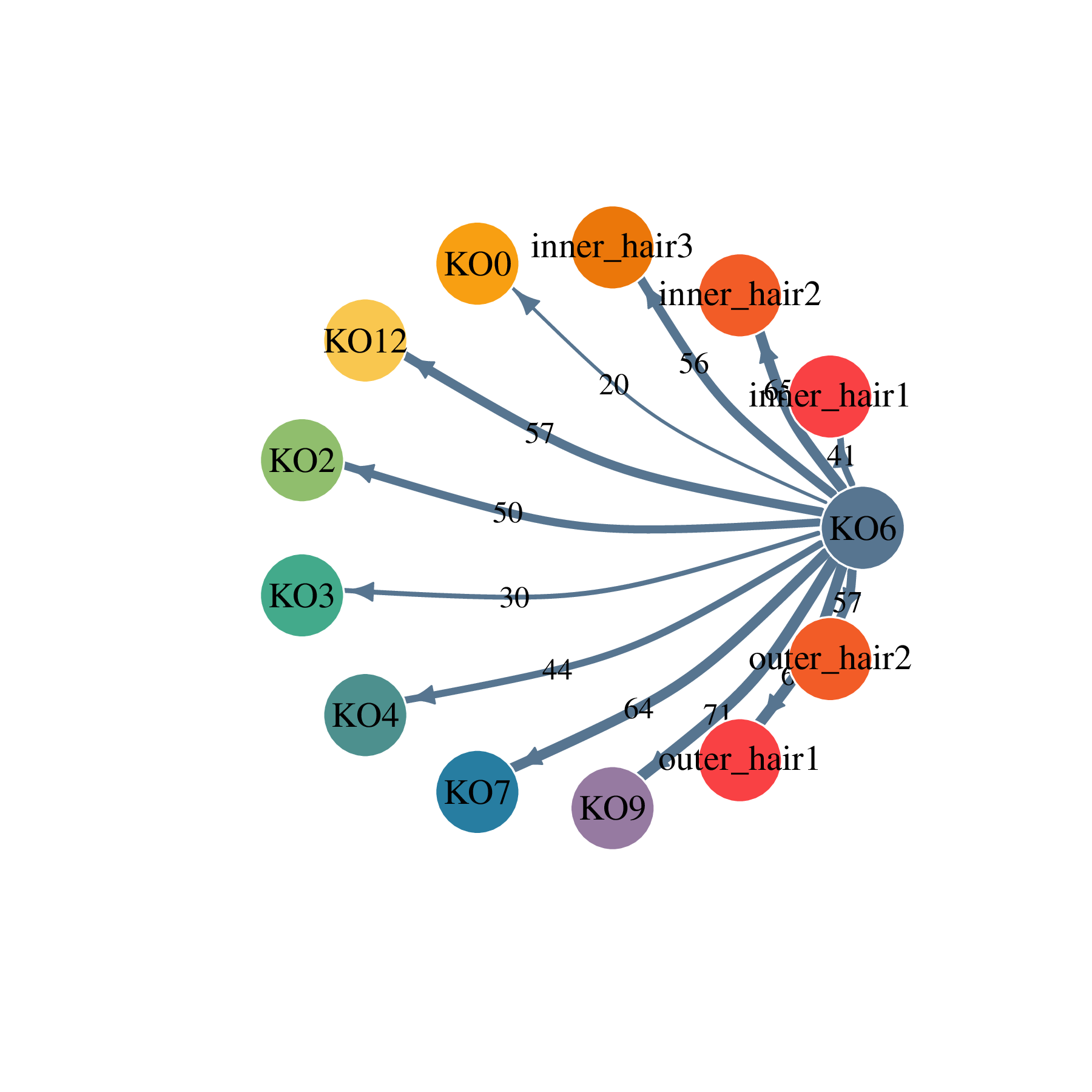

Supplement: Supplementary file 20 [file Image_8.PNG]

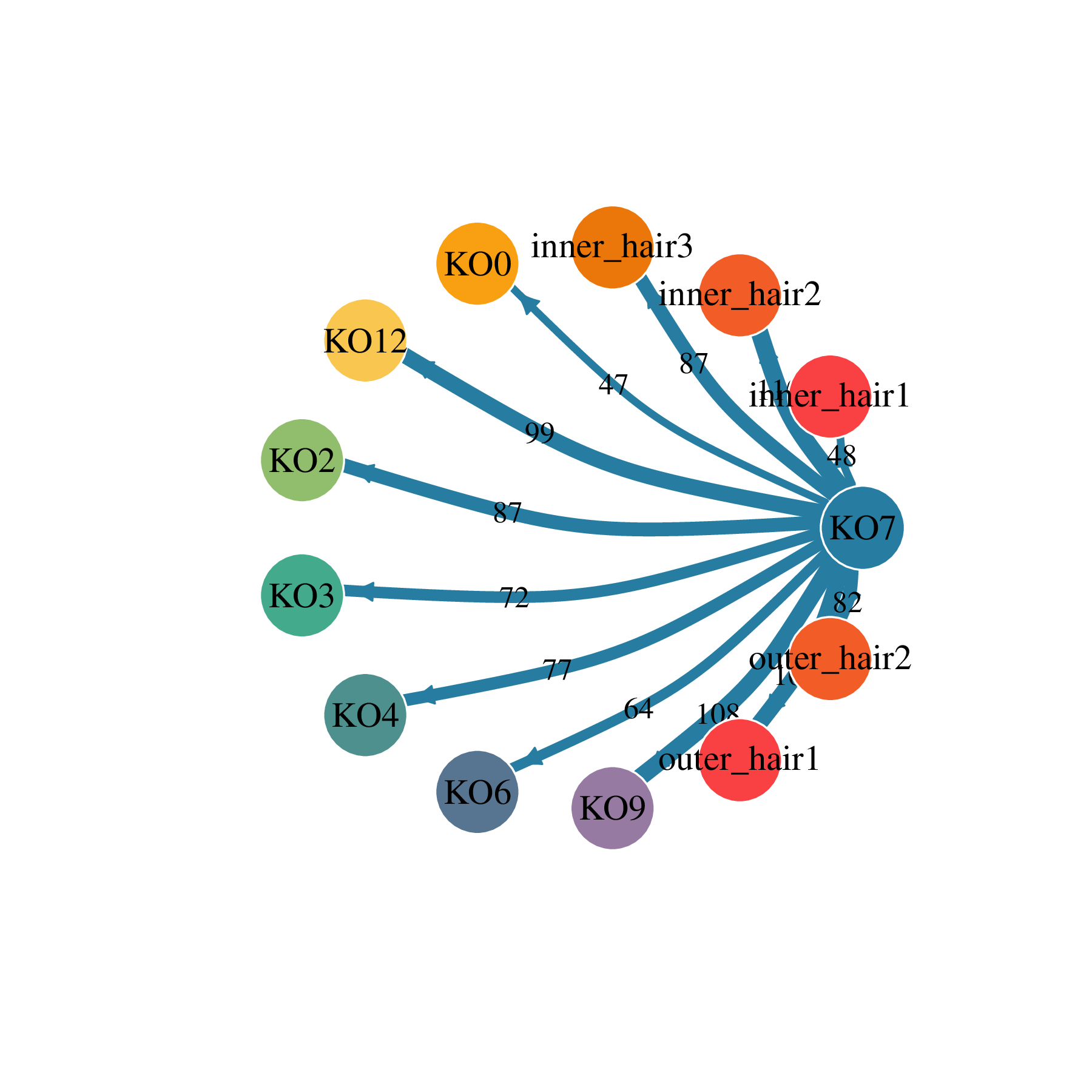

Supplement: Supplementary file 21 [file Image_9.PNG]

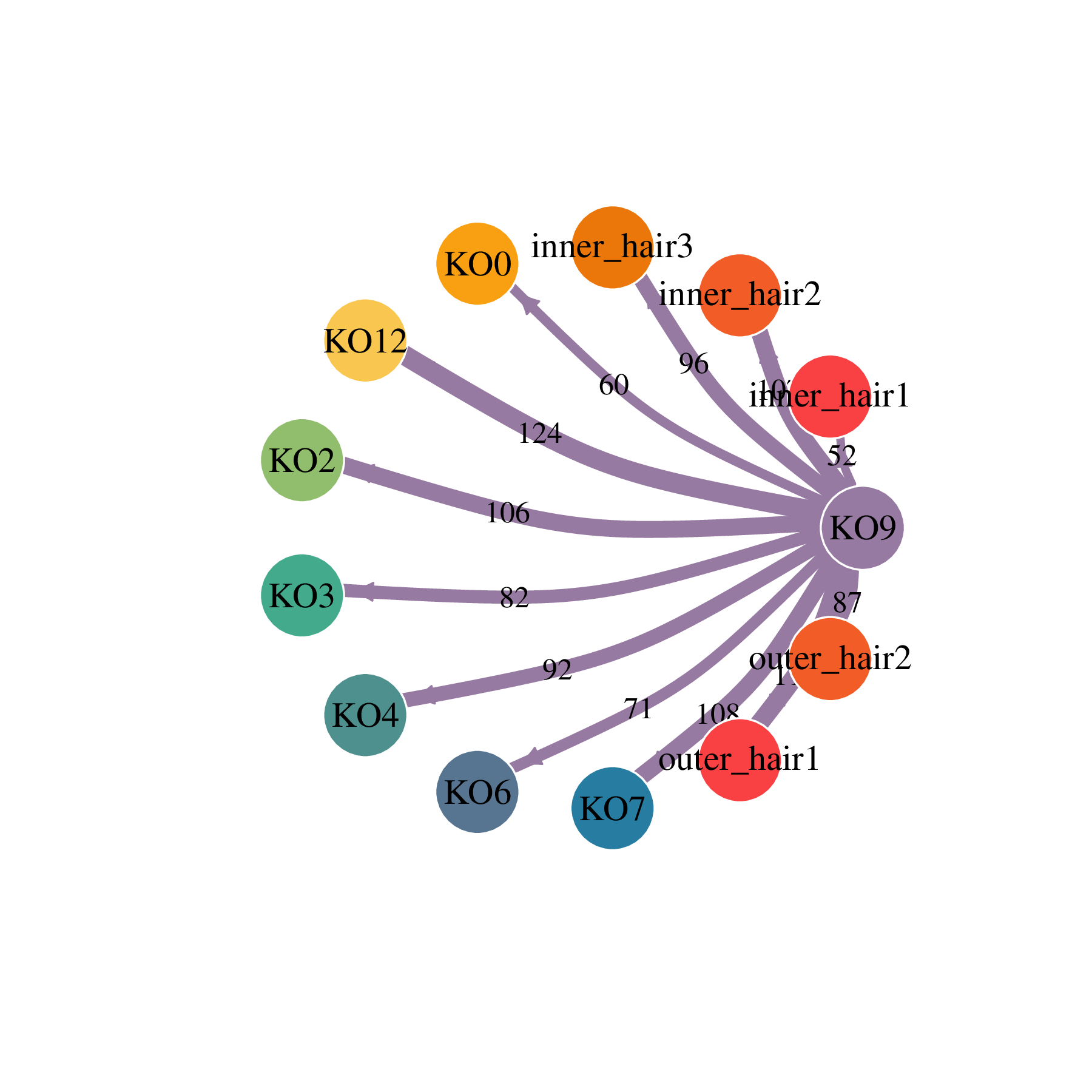

Supplement: Supplementary file 22 [file Image_10.PNG]

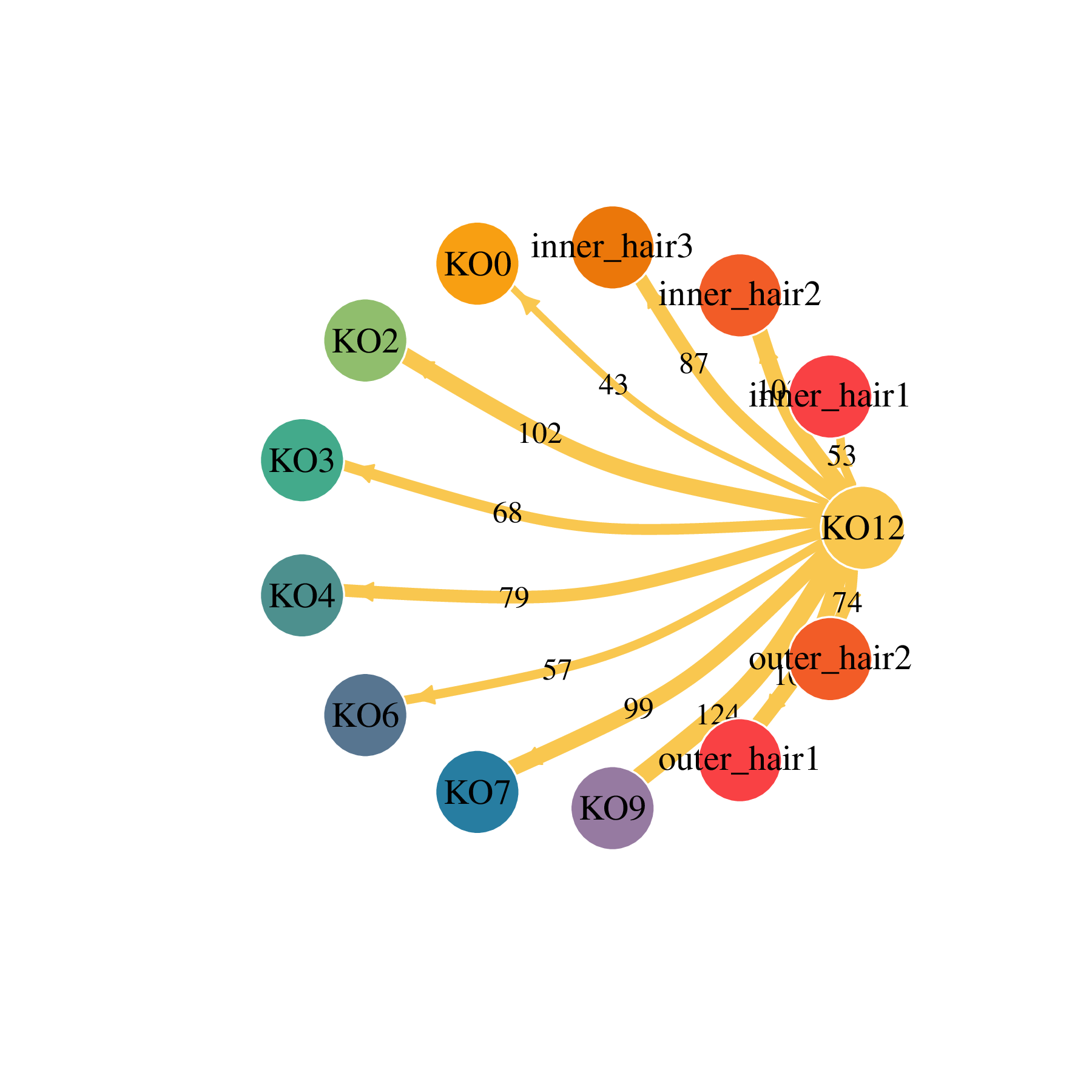

Supplement: Supplementary file 23 [file Image_11.PNG]

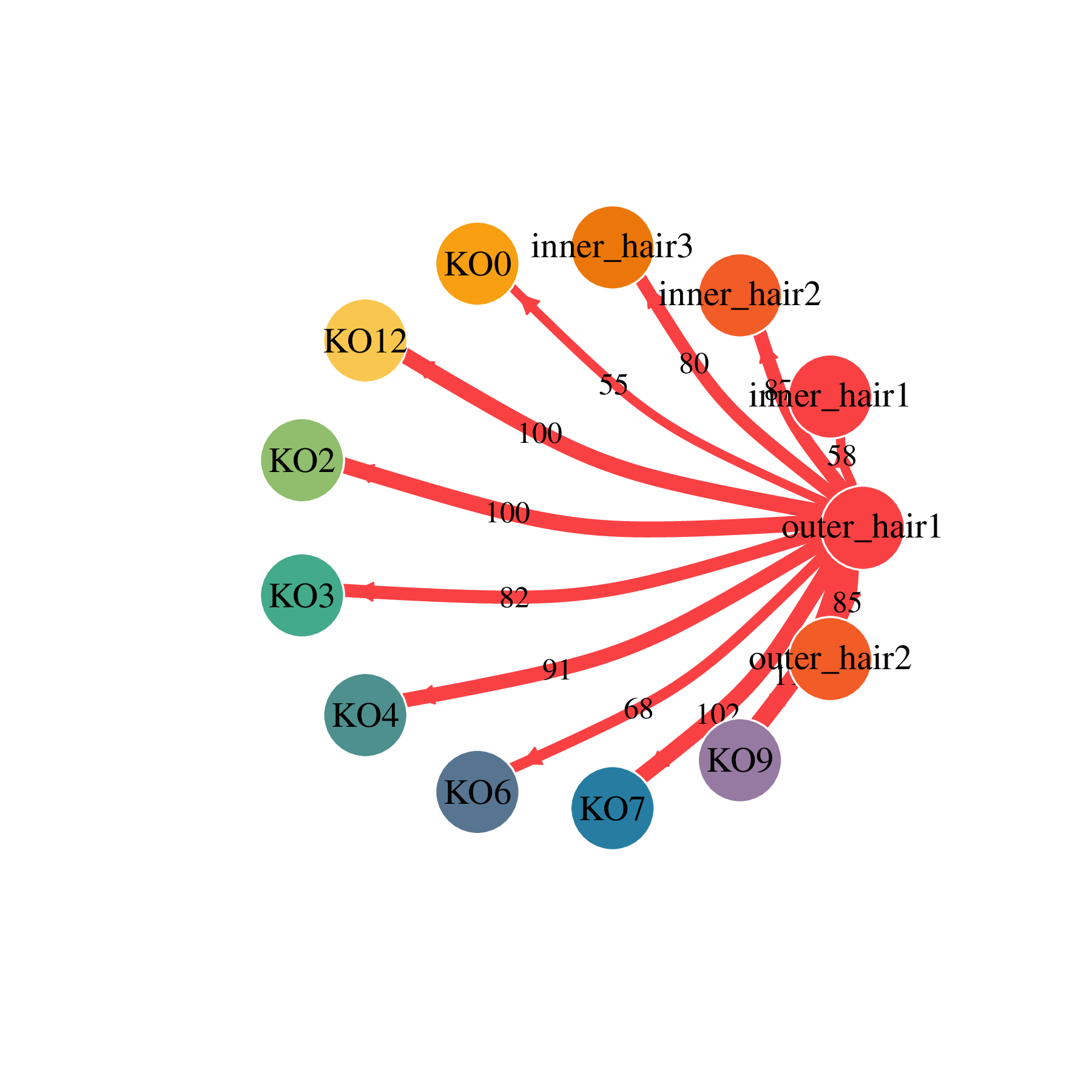

Supplement: Supplementary file 24 [file Image_12.PNG]

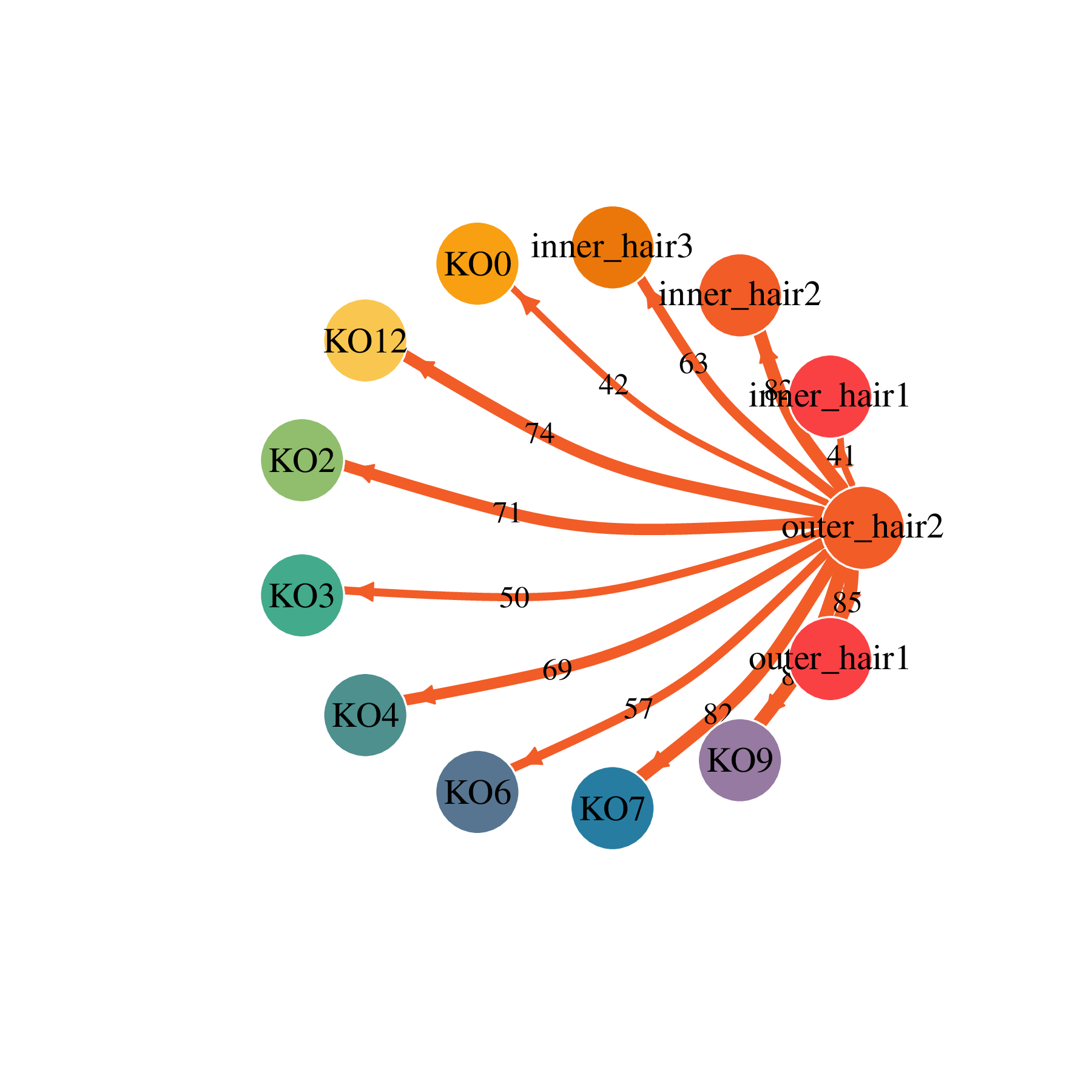

Supplement: Supplementary file 25 [file Image_13.PNG]
